# The SmNPR4-SmTGA5 module regulates SA-mediated phenolic acid biosynthesis in *Salvia miltiorrhiza* hairy roots

**DOI:** 10.1093/hr/uhad066

**Published:** 2023-04-10

**Authors:** Meiling Ding, Bin Zhang, Shuo Zhang, RongRong Hao, Yu Xia, Pengda Ma, Juane Dong

**Affiliations:** College of Life Sciences, Northwest A&F University, Yangling 712100, China; College of Life Sciences, Northwest A&F University, Yangling 712100, China; College of Life Sciences, Northwest A&F University, Yangling 712100, China; College of Life Sciences, Northwest A&F University, Yangling 712100, China; College of Life Sciences, Northwest A&F University, Yangling 712100, China; College of Life Sciences, Northwest A&F University, Yangling 712100, China; College of Life Sciences, Northwest A&F University, Yangling 712100, China

## Abstract

Phenolic acids are the main bioactive compounds in *Salvia miltiorrhiza*, which can be increased by salicylic acid (SA) elicitation. However, the specific molecular mechanism remains unclear. The nonexpresser of PR genes 1 (NPR1) and its family members are essential components of the SA signaling pathway. Here, we report an NPR protein, SmNPR4, that showed strong expression in hairy root after SA treatment, acting as a negative moderator of SA-induced phenolic acid biosynthesis in *S. miltiorrhiza* (*S. miltiorrhiza*). Moreover, a basic leucine zipper family transcription factor SmTGA5 was identified and was found to interact with SmNPR4. SmTGA5 activates the expression of phenolic acid biosynthesis gene *SmTAT1* through binding to the *as-1* element. Finally, a series of biochemical assays and dual gene overexpression analysis demonstrated that the SmNPR4 significantly inhibited the function of SmTGA5, and SA can alleviate the inhibitory effect of SmNPR4 on SmTGA5. Overall, our results reveal the molecular mechanism of salicylic acid regulating phenolic acid biosynthesis in *S. miltiorrhiza* and provide new insights for SA signaling to regulate secondary metabolic biosynthesis.

## Introduction

*Salvia miltiorrhiza* (*S. miltiorrhiza*) is a very popular traditional herb in China, which can treat various diseases, mainly with the dried root or rhizome [1]. Hydrophilic phenolic acids are the primary active components, including rosmarinic acid (RA), salvianolic acid B (Sal B) and caffeic acid (CA), which have significant efficacy on treating cardiovascular disease, with the virtue of endothelial protective, anti-inflammatory, and anti-atherosclerosis [1, 2]. The phenolic acid biosynthesis involves two parallel pathways: the tyrosine-derived pathway and the phenylpropanoid pathway. The products of these upstream pathways undergo condensation reactions to form RA, followed by other reactions to form different salvianolic acids, like Sal B^3^. The biosynthesis enzymes of phenolic acids have been identified and analysed, such as tyrosine aminotransferase (TAT), 4-hydroxyphenylpyruvate reductase (HPPR), phenylalanine ammonia-lyase (PAL), cinnamic acid 4-hydroxylase (C4H), 4-coumarate: CoA ligase (4CL), rosmarinic acid synthase (RAS), and CYP98A14, etc. [3–6].

Because of the low yield of bioactive ingredients in medicinal plants, they cannot satisfy the rapidly growing market demand. Hence, improving phenolic acid content through biotechnology approaches, such as elicitation treatment, hairy root culture, transcription regulation and synthetic biology is significant [6]. Salicylic acid (SA) acts as a vital hormone that regulates many plant responses, especially plant immunity against pathogens [7–10]. Moreover, SA stimulates the accumulation of numerous active components in medicinal plants. For example, SA treatment increased the yield of taxol in *Taxus chinensis* [11], artemisinin in *Artemisia annua* L [12, 13], and phenolic acids in *S. miltiorrhiza* [14, 15]. However, the regulatory mechanism of SA-induced biosynthesis of secondary metabolites remains unclear.

The NONEXPRESSOR OF PATHOGENESIS-RELATED GENE1 (NPR1), a pivotal protein in the SA signaling pathway, has been proven to be an SA receptor with important significance in SA-mediated plant immunity [16–19]. As paralogs of NPR1, *Arabidopsis* NPR3 and NPR4 share high homology with NPR1 and have also been shown to function as SA receptors [17, 20–23]. Although NPR1/3/4 are all associated with pathogen defense reactions, only NPR1 plays a positive effect in SA-induced systemic acquired resistance (SAR) [17, 24]. Early research showed that NPR4 and NPR3 act as adaptors of E3 ligase, mediating the degradation of NPR1 in an SA-regulated manner [21]. However, recent studies have demonstrated that NPR3/4 act as transcriptional co-repressors, and their roles are distinct and separate from NPR1 [17, 25]. SA induces the expression of immune genes by suppressing the activity of NPR3/4 [17].

Numerous studies have reported that transcription factors (TFs) such as bHLHs, bZIPs, WRKYs, and MYBs are involved in regulating phenolic acid biosynthesis [26–30]. However, the association between TFs and SA-induced phenolic acid biosynthesis has been poorly studied. Through their binding to TGACG elements in the activation sequence-1 (*as-1*), TGAs, a type of basic leucine zipper (bZIP) TFs, act as vital proteins in the SA signaling pathway and can interact with NPRs to mediate this process [17, 31, 32]. In *Arabidopsis*, ten TGAs have been classified into five clades [33]. Among them, the clade II TFs (TGA2/5/6) were studied the most, which have functional redundancy and form enhancer complexes with NPR1 proteins to participate in the plant pathogen defense response [34, 35]. In addition, TGAs also regulate plant secondary metabolism. For example, overexpression of *TwTGA1* increased the production of triptolide and two alkaloids in *T. wilfordii* Hook. f. cells [36], AaTGA6 promoted artemisinin accumulation by activating the transcriptional expression of *AaERF1* in *A. annua* [13], and the expression of natural rubber biosynthesis enzyme genes were regulated by *HbTGA1* in *Hevea brasiliensis* [37].

**Figure 1:**
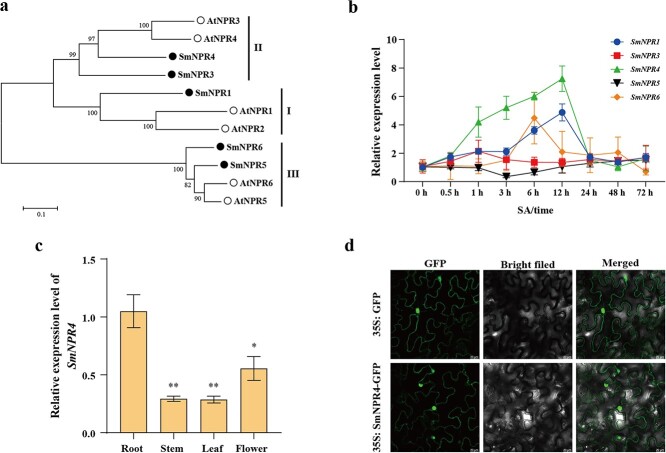
SmNPR4 are the candidate genes for Sal B biosynthesis. **a** The evolutionary tree of SmNPRs and NPRs from *Arabidopsis thaliana*. **b** The transcript levels of the *SmNPR* genes were measured after 50 μM SA treatment in wild hairy roots. **c** The transcript levels of the *SmNPR4* from roots, leaves, flowers, and stems tissues of 2-year-old *Salvia miltiorrhiza* were measured by qRT-PCR. Bars indicate means ±SD (*n* = 3, Student *t* tests, ^*^P < 0.05 and ^**^P < 0.01). **c** Subcellular localization analysis of the SmNPR4 protein. The GFP signal was used as the negative control.

In this study, we examined the functions of SmNPR4 in *S. miltiorrhiza* and found that it has negative roles in SA-induced salvianolic acid biosynthesis. Subsequently, we identified a key TF, SmTGA5, which interacts with SmNPR4. SmTGA5 is a positive regulator of salvianolic acid biosynthesis, where *SmTAT1* is a target gene of SmTGA5. The transcriptional activation of SmTGA5 was found to be repressed by SmNPR4, and SA can relieve this repression. In conclusion, our study reveals the molecular mechanism of the SmNPR4-SmTGA5 module in SA-mediated phenolic acid biosynthesis.

**Figure 2:**
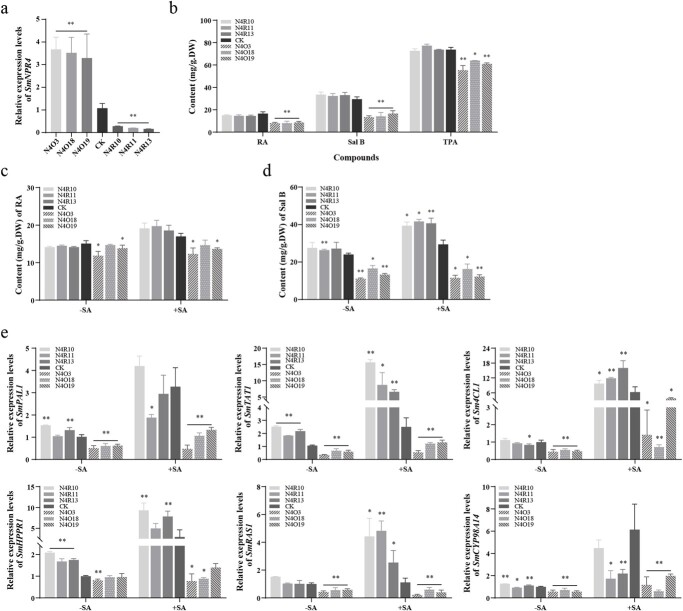
The effect of SmNPR4 in the accumulation of phenolic acid biosynthesis and the expression of biosynthetic genes induced by SA. **a** qRT-PCR analysed the transcript level of *SmNPR4* in the CK, N4O and N4R lines. **b** The phenolic acid content of CK, N4O, and N4R lines were detected by HPLC and Folin–Ciocalteu method. The changes of RA (**c**) and Sal B (**d**) content in the transgenic lines and control following with (+) or without (−) SA treatment. After 18 days of simultaneous culture, the CK, N4O, and N4R lines were treated with 50 μM SA for 3 days. **e** qRT-PCR analysed the transcript levels of biosynthetic genes in all lines with (+) or without (−) SA treatment. Bars indicate means ±SD (*n* = 3, Student *t* tests, ^*^P < 0.05 and ^**^P < 0.01).

## Results

### *SmNPR4* is the candidate gene associated with SA-mediated phenolic acid biosynthesis

Previous works have shown that accumulation of salvianolic acid and the expression of biosynthetic genes could be induced after SA treatment [14, 15]. As the SA receptors, NPR proteins are indispensable components of SA signaling pathway [7]. To identify NPRs associated with SA-induced phenolic acid biosynthesis, we used HMMER to search the *S. miltiorrhiza* genomic database and identified five SmNPR proteins. Those proteins were named SmNPR1, SmNPR3, SmNPR4, SmNPR5, and SmNPR6 according to the nomenclature of NPRs in *Arabidopsis thaliana (*A. thaliana*)* (Table S2, see online supplementary material). The phylogenic tree analysis classifies the NPRs from *S. miltiorrhiza* and *A. thaliana* into three subgroups (Fig. 1a). We then measured the transcript levels of *SmNPRs* in wild hairy roots with post 50 μM SA treated at different time intervals. SmNPR4 expression increased 6.2-fold after 12 h of SA induction, showing the highest response to SA (Fig. 1b). Based on the above results, SmNPR4 was selected for further research.

The coding sequence of *SmNPR4* contained a 1776 bp open reading frame (ORF), encoding a 591 amino acids protein. According to the multiple alignment of the amino acid sequence, SmNPR4 had high similarity with AtNPR3/4, and the C-terminal region has putative EAR motif (Fig. S1, see online supplementary material). The tissue expression analysis indicated that *SmNPR4* is highly expressed in the roots comparing with the other tissues like stems, leaves, and flowers (Fig. 1c). To determine the subcellular localization of SmNPR4, we transformed recombinant vectors containing SmNPR4-GFP fusion protein and GFP alone into *Agrobacterium* strain GV3101. As Fig. 1d showed, SmNPR4 signal were mainly distributed in the nucleus but also present in the cytoplasm, implying that SmNPR4 may be involved in transcriptional regulation.

### SmNPR4 is a negative moderator in SA-induced phenolic acid accumulation

To determine the effect of SmNPR4 in the phenolic acid biosynthesis, the *SmNPR4* overexpression and RNA interference transgenic hairy roots were obtained. We then obtained positive transgenic hairy roots by genomic PCR identification. The *NPR4*-overexpression lines (N4O) showed positive rate was 70%, and the *NPR4* RNAi lines (N4R) showed positive rate was 50% (Fig. S2, see online supplementary material). Hairy roots cultured ATCC15834 without plasmid were used as control (CK). Through qRT-PCR analysis, we obtained three N4O lines (N4O3, N4O18, N4O19) with high *SmNPR4* expression and three N4R lines (N4R10, N4R11, N4R13) with low *SmNPR4* expression for next study (Fig. 2a).

The content of phenolic acids in CK, N4O, and N4R lines by using high-performance liquid chromatography (HPLC) assay. The RA and Sal B content of the N4O lines were obviously lower than those of the CK, and the Sal B content in the N4O lines was 46% to 56% of that in the CK [29.4 mg/g dry weight (DW)]. Moreover, the yield of total phenolic acids (TPA) was also examined in these lines by the Folin–Ciocalteu method, and the trend was consistent with the HPLC analysis (Fig. 2b). To investigate whether SmNPR4 plays a role in the SA-mediated salvianolic acid biosynthesis pathway, we examined the changes of RA and Sal B content in the CK, N4O and N4R lines after SA induction. The average increase in content of RA and Sal B in N4R lines were 1.19-fold and 1.23-fold of that in CK after SA treatment, respectively. On the contrary, the average increase in content of RA and Sal B in N4O lines were 0.89-fold and 0.80-fold of that in CK after SA treatment, respectively (Fig. 2c and d). These results indicate that SmNPR4 has a major role in the SA-mediated salvianolic acid accumulation pathway.

**Figure 3:**
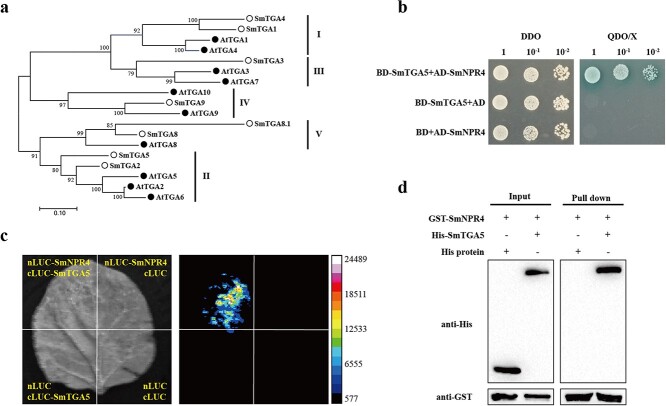
Interactions between SmNPR4 and SmTGA5. **a** Phylogenic tree analysis of the TGAs from *Salvia miltiorrhiza* and *Arabidopsis thaliana*. **b** Y2H assay showed that the interaction between SmNPR4 and SmTGA5. DDO, SD/−Leu/−Trp medium; QDO/X, SD/−Leu/−Trp/−Ade/-His with x-α-gal medium. **c** LCI assay confirmed that the interaction of SmNPR4 and SmTGA5 in tobacco leaves. **d** The interaction of SmNPR4 and SmTGA5 detected by pull-down assays. The His-SmTGA5 and His proteins was incubated, respectively, with GST-SmNPR4. Proteins that were pulled down with GST beads were identified by using anti-His and anti-GST antibodies.

### SmNPR4 affects the expression of enzyme genes related to salvianolic acid biosynthesis

To further expound the function of SmNPR4 in SA-mediated salvianolic acid biosynthesis, we detected the expression of the enzyme genes in transgenic lines with and without SA treatment by qRT-PCR (Fig. 2e). The expression of the *SmPAL1*, *SmTAT1*, *Sm4CL1*, *SmRAS1*, and *SmCYP98A14* genes was markedly reduced in the N4O lines without SA treatment. Following SA treatment, most genes were upregulated in all lines, except for *SmRAS1* in some lines. However, N4O lines showed lower average increases in gene expression compared to CK. For instance, the average increase in expression of *SmPAL1*, *SmTAT1*, and *SmCYP98A14* gene in N4O lines were 51%, 81%, and 33% of that in CK after SA treatment, respectively. In contrast, N4R lines exhibited higher average increases in *Sm4CL1*, *SmTAT1*, and *SmRAS1* expression than CK after SA treatment, with increases of 2.04-fold, 2.03-fold, and 2.97-fold, respectively. These findings further demonstrate that SmNPR4 is participated in SA-mediated phenolic acid biosynthesis and plays a negative regulatory role.

### A bZIP transcription factor SmTGA5 interacts with SmNPR4

It is well known that NPRs lack a DNA-binding domain and can’t directly regulate the transcriptional expression of target genes. The clade II TGA members (TGA2/5/6) have been reported to interact with NPR proteins to synergistically regulate the expression of downstream target genes [22, 31, 32]. Analysis of the enzyme promoter sequences showed that many *as-1* elements are present in these DNA segments, implying that TGA TFs may be involved in the Sal B biosynthesis (Fig. S3, see online supplementary material).

A total of eight SmTGA proteins were identified by searching *S. miltiorrhiza* genomic data using HMMER (Table S2, see online supplementary material). A phylogenetic tree was constructed based on SmTGAs and 10 AtTGAs from *Arabidopsis*. The result showed that all TGAs were divided into five clades. SmTGA2/5 shared a close evolutional relationship with AtTGA2/5/6 and were classified into the subgroup II (Fig. 3a). To explore which TGA is associated with phenolic acid biosynthesis, we constructed a clustering heat map containing enzyme genes and *SmTGAs* based on several RNA-seq data (Fig. S4, see online supplementary material). Based on the expression profile of these genes, three main groups were divided. In group A, only *SmTGA5* was located in the same large cluster with the 12 enzyme genes, suggesting that this TGA most likely regulates phenolic acid biosynthesis. We speculated that SmTGA5 might interact with SmNPR4 protein and therefore performed a Y2H assay. The Y2H assay showed that only Y2H yeast cells co-transformed by BD-SmTGA5 and AD-SmNPR4 grow well on the QDO/X selective medium, but others can’t grow (Fig. 3b) We next used LCI assay to verify this interaction in planta. The result showed that fluorescence was detected only in the area co-transformed with nLUC-SmNPR4 and cLUC-SmTGA5 in leaves of **Nicotiana benthamiana* (*N. benthamiana*)*, indicating that SmNPR4 interacted with SmTGA5 *in vivo* (Fig. 3c). In addition, the *in vitro* interaction between SmNPR4 and SmTGA5 was confirmed by pull-down assay. GST-SmNPR4 interacted with His-SmTGA5 but not with His control (Fig. 3d). These results indicate that SmNPR4 interacted with SmTGA5.

### Characteristic of *SmTGA5*

The ORF of *SmTGA5* was 1377 bp and encoded a 458 amino acid protein. The subcellular localization of SmTGA5 was checked, and the results displayed that the GFP signal of control was present in both cytoplasm and nucleus, while the GFP signal of SmTGA5 was detected only in the nucleus, indicating that SmTGA5 functions as a TF (Fig. S5, see online supplementary material).

To confirm the tissues expression patterns of *SmTGA5*, we examined the transcript levels in different tissues of *S. miltiorrhiza*. It was found that *SmTGA5* was extensively expressed in all tissues, peak expression in roots (Fig. S6a, see online supplementary material). To investigate whether the expression of *SmTGA5* was affected by SA, the expression level of *SmTGA5* after 50 μM SA treatment was detected by qRT-PCR. The SA upregulated the expression of *SmTGA5*, and the peak was reached at 12 h (Fig. S6b, see online supplementary material).

### SmTGA5 positively affects phenolic acid biosynthesis

**Figure 4:**
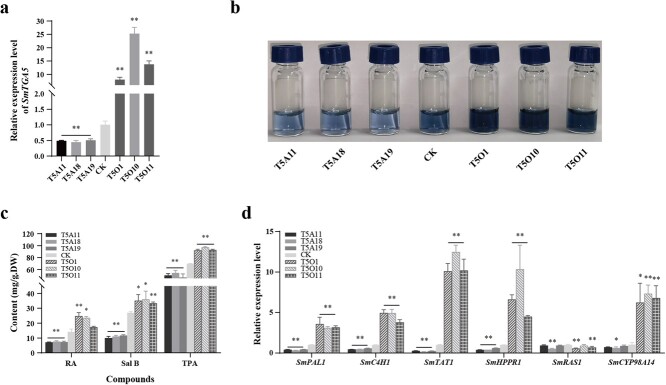
Effects of SmTGA5 on phenolic acid content and enzyme genes expression. (a) The expression level of *SmTGA5* was measured in the CK, T5O and T5A lines by using qRT-PCR. (b) After staining total phenolic acid extracts from the CK, T5O and T5A lines with the Folin–Ciocalteu method, qualitative analyses were performed. (c) phenolic acid content in transgenic lines and CK detected by HPLC and Folin–Ciocalteu method. (d) The transcript levels of enzyme genes were detected in the CK, T5O and T5A lines by using qRT-PCR. Bars indicate means ±SD (n = 3, Student t-tests, ^*^P < 0.05 and ^**^P < 0.01).

To confirm the function of SmTGA5 in phenolic acid biosynthesis, transgenic hairy roots were generated by overexpression and antisense expression strategies, respectively. We then obtained positive lines by genomic PCR identification (Fig. S7, see online supplementary material). Whereafter, qRT-PCR analysis revealed that the expression of *SmTGA5* in the T5O lines (T5O1, T5O10, T5O11) was 8 to 25-fold higher than CK, while the transcript level of *SmTGA5* was lower in the T5A lines (T5A11, T5A18, T5A19) than in CK (Fig. 4a), so those transgenic lines were used for next researches.

Using the Folin–Ciocalteu method, we qualitatively determined the total phenolics content in CK and transgenic hairy roots by observing color changes. T5O lines showed significantly darker colors than CK, indicating that overexpressing SmTGA5 increased the total phenolics content. In contrast, T5A lines had lighter colors (Fig. 4b). Correspondingly, the TPA content in the transgenic lines was consistent with its color change trend. The content of salvianolic acids in CK, T5O and T5A lines were determined by HPLC. The results indicated that the Sal B content was markedly increased in the T5O lines than the CK, with a variation range of 33.36 to 35.98 mg/g DW. Conversely, the average content of RA and Sal B in T5A lines declined to 7.39 mg/g DW and 10.90 mg/g DW, respectively, compared to CK (Fig. 4c). The promoter of salvianolic acid biosynthetic pathway genes including *SmTAT1*, *SmHPPR1*, *SmPAL1*, *SmC4H1*, *SmRAS1*, and *SmCYP98A14* all contain *as-1* elements (Fig. S3, see online supplementary material), so we examined expression of these genes in in the CK, T5O, and T5A lines. The expression of *SmTAT1*, *SmHPPR1*, *SmPAL1*, *SmC4H1*, and *SmCYP98A14* showed obvious upregulation in the T5O lines more than the CK. Conversely, the transcript levels of these enzyme genes were more decreased in the T5A lines than the CK (Fig. 4d). These results suggest that SmTGA5 has a positive regulatory function in phenolic acids biosynthesis.

### SmTGA5 directly activates the expression of *SmTAT1* gene *in vivo* and *in vitro*

**Figure 5:**
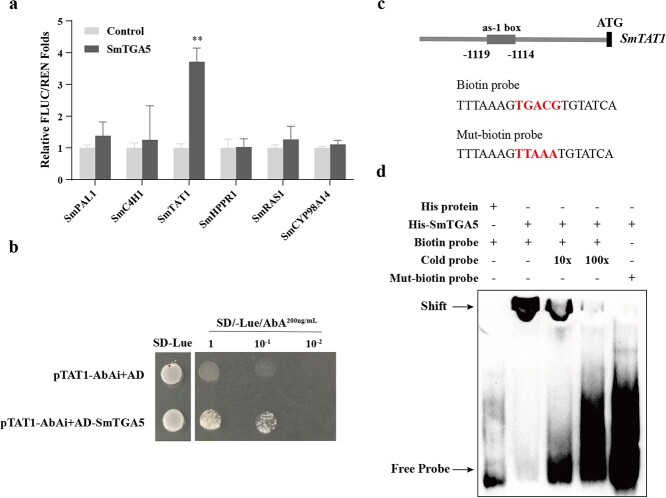
SmTGA5 can bind and activate the *SmTAT1* promoter. **a** Dual-LUC analysis examined the effect of SmTGA5 on the expression of biosynthetic genes. Bars indicate means ±SD (*n* = 3, Student *t* tests, ^*^P < 0.05 and ^**^P < 0.01). **b** Y1H assay showed the interactions between SmTGA5 and the promoter of SmTAT1, pTAT1-AbAi+pGADT7 as the negative control. **c** The design of probes containing the *as-1* box region in the SmTAT1 promoter for EMSA experiments. The biotin probe including the TGACG sequence and the mut-biotin probe including the TTTAA sequence were used in EMSA assay. **d** EMSA analysis indicated that His-SmTGA5 specifically binds the *as-1* box in the *SmTAT1* promoter.

To examine how SmTGA5 regulates phenolic acid biosynthesis, a Dual-LUC assay was performed. The results indicated that SmTGA5 markedly enhanced the expression of *SmTAT1* by 4-fold, whereas mutation of the *as-1* element in the *SmTAT1* promoter significantly reduced the expression (Fig. 5a; Fig. Figure S8, see online supplementary material). Y1H analysis revealed that SmTGA5 bound to the *SmTAT1* promoter which contains *as-1* element (Fig. 5b). In addition, the Electrophoretic mobility shift assay (EMSA) result also verified the DNA-binding activity of SmTGA5. The retarded bands of the complex containing protein and probe were observed exclusively in the existence of His-SmTGA5 fusion protein. While increasing the concentration of unlabeled probes, the concentration of retarded bands decreased. When “TGACG” was mutated to “TTAAA” in the sequence, SmTGA5 was unable to bind to the mutated probe (Fig. 5c and d). These findings suggest that SmTGA5 specifically recognizes the *as-1* box in the *SmTAT1* promoter sequence.

### The function of SmTGA5 was inhibited by SmNPR4 in phenolic acid biosynthesis

**Figure 6:**
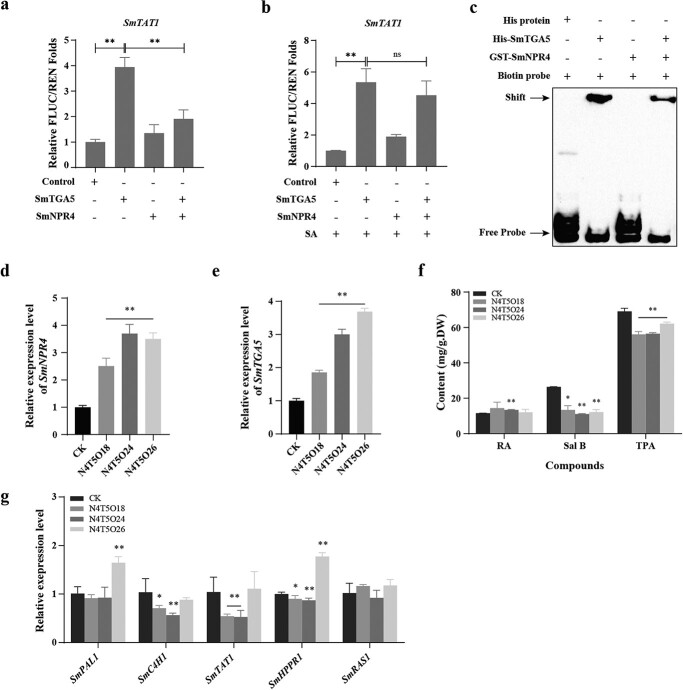
The role of SmTGA5 was inhibited by SmNPR4 in phenolic acid biosynthesis. **a** Dual-LUC assay for expression levels of reporter genes in tobacco leaves with SmTGA5 alone or with SmNPR4 and SmTGA5 co-expressed. **b** The reporter expression levels of SmTGA5 alone or SmNPR4 and SmTGA5 co-expressed in tobacco leaves treated with 0.5 M SA for 6 h were determined by Dual-LUC assay. **c** The EMSA analysis shows that the activity of SmTGA5 binding to *SmTAT1* promoter is suppressed by SmNPR4. The expression level of *SmTGA5* (**d**) and *SmNPR4* (**e**) in the N4T5O lines. **f** Measurement of phenolic acid content in CK and N4T5O lines. **g** The expression of enzyme genes in N4T5O lines detected by qRT-PCR. Bars indicate means ±SD (*n* = 3, Student *t* tests, ^*^P < 0.05 and ^**^P < 0.01).

Based on our data, SmNPR4 and SmTGA5 might form a complex to regulate the expression of *SmTAT1*. Therefore, Dual-LUC assay and EMSA assay were utilized to confirm this hypothesis. The results revealed that the transcriptional level of *SmTAT1* was attenuated when SmNPR4 and SmTGA5 were co-present (Fig. 6a). In addition, the relative FLUC/RLUC folds of *SmTAT1* in the co-presence of SmNPR4 and SmTGA5 were not significantly different from those of *SmTAT1* in the presence of only SmTGA5 after SA treatment, indicating that SA treatment could alleviate this inhibition (Fig. 6b). In further, SmNPR4 was found to attenuate the DNA-binding activity of SmTGA5 by EMSA assay (Fig. 6c).

To further elucidate the function of SmNPR4-SmTGA5 module *in vivo*, we constructed *SmNPR4* and *SmTGA5* double overexpression vectors and performed genetic transformation to obtain transgenic hairy roots (Fig. S9a, see online supplementary material). The positive lines were obtained by genomic PCR identification (Fig. S9b, see online supplementary material). The expression levels of *SmNPR4* and *SmTGA5* in the N4T5O lines (N4T5O18, N4T5O24, N4T5O26) were detected by qRT-PCR. It was observed that the N4T5O lines exhibited a significant increase in the expression levels of *SmNPR4* and *SmTGA5*. (Fig. 6d and e). Subsequently, we examined the phenolic acid content in the N4T5O lines. The RA content was higher in the N4T5O lines than CK, but the content of Sal B and TPA were significantly decreased, with the average content of Sal B decreasing to 12.20 mg/g DW (Fig. 6f). Besides, we also detected the transcript levels of some biosynthetic genes in those lines. The transcript levels of *SmPAL1* and *SmHPPR1* genes were higher in the N4T5O26 line than CK, and the transcript levels of *SmTAT1, SmHPPR1*, and *SmC4H1* genes were much lower in other lines than CK (Fig. 6g). In a word, these findings suggest that SmNPR4 inhibits the function of SmTGA5 in regulation of phenolic acid biosynthesis.

## Discussion

SA is an essential plant defense hormone that plays a vital role in broad-spectrum plant immunity [7]. Earlier studies have revealed that SA increases *SmPAL1* and *SmTAT1* gene expression and related enzyme activities, thereby promoting phenolic acid accumulation in *S. miltiorrhiza* cells [14, 15]. Our findings corroborate previous research, indicating that SA treatment enhanced the content of RA and Sal B in *S. miltiorrhiza*. Furthermore, the upregulation of *SmPAL1* and *SmTAT1* genes was also observed (Fig. 2). However, the potential mechanism of SA elicitation is still unclear.

The NPR1/3/4 proteins are SA receptors, with NPR4 having the strongest binding affinity for SA [17, 23]. Here, the expression of SmNPR4 was dramatically increased after SA induction (Fig. 1b). In *Arabidopsis*, *NPR4* is also responsive to SA, but it functions as a transcriptional co-repressor in plant immunity [17, 22], implying that NPR4 may be involved in the negative feedback regulation of SA signaling, reducing the output of SA signaling. NPR4 has a similar structural domain with NPR1, but their function is opposite in the transcription regulation of plant immune [17]. The C-terminal sequence of NPR4 has a conserved motif (VDLNETP) that is highly similar to the EAR motif, which is essential for the transcriptional repressing activity of SmNPR4 on downstream target genes [17]. The putative EAR motif was found in SmNPR4 (Fig. S1, see online supplementary material), indicating that SmNPR4 may have transcriptional repressing activity. SmNPR4-GFP accumulated in both the nucleus and cytoplasm of tobacco, which differed from the localization of AtNPR4-GFP [18, 20], suggesting that SmNPR4 may have other functions besides transcriptional regulation.

Overexpression of *SmNPR4* decreased salvianolic acids content and key enzyme genes expression levels, whereas RANi of *SmNPR4* did not affect phenolic acid biosynthesis (Fig. 2b), possibly due to the functional redundancy of NPR3 with NPR4 [17, 22]. The N4R lines showed the greatest changes in RA and Sal B content after SA treatment, followed by CK. Correspondingly, the expression of some enzyme genes changed similarly to the changes in phenolic acid content after SA treatment (Fig. 2c–e). These results demonstrate that SmNPR4 is involved in SA-induced phenolic acid biosynthesis and acts a negative moderator of this pathway. However, as a negative moderator, how to inhibit SmNPR4 function after SA treatment remains unknown. Although the crystal structure and key amino acid residues of AtNPR4 protein binding to SA have been reported, it is still unknown how SA changes the conformation of NPR4 and inhibits its activity [23]. Therefore, further studies are needed to understand the mechanisms of how SA changes the activity of the NPR4 protein.

In plants, TGA TFs regulate the *PR-1* gene expression by interacting with the non-DNA-binding protein NPRs, which together constitute an important part of the SA-controlled signaling cascade [33]. Here, eight TGA TFs with complete structural domains were screened in *S. miltiorrhiza* and found that *SmTGA5* was clustered with most enzyme genes by clustering heat maps, which includes the *SmTAT1* gene (Fig. S4, see online supplementary material). The expression of *SmNPR4* and *SmTGA5* genes were the highest in root tissues, and their subcellular localization was similar, suggesting that SmNPR4 and SmTGA5 were functionally related. In *Arabidopsis*, NPR3/4 interacts with Class II TGA TF to exert a negative regulatory effect on the expression of target genes involved in pathogen resistance [22]. The interaction between SmTGA5 and SmNPR4 was also confirmed by Y2H, LCI, and pull-down assays (Fig. 3).

SmTGA5 belongs to subgroup II TGA TFs. In *Arabidopsis*, members of this subgroup have functional redundancy and are capable of positively regulating SA-induced *PR-1* gene expression [34]. Therefore, we guessed that SmTGA5 may have a positive regulatory effect in phenolic acid biosynthesis mediated by SA. Our results found that overexpression of *SmTGA5* led to a notable increase in phenolic acid production. Conversely, a decrease in phenolic acid production was observed in the T5A lines (Fig. 4b and c). Correspondingly, the expression of other genes except for *SmRAS1* was notably upregulated in T5O lines, among them *SmTAT1* had the highest expression level (Fig. 4e). Dual-LUC assays showed that SmTGA5 markedly activated *SmTAT1* expression (Fig. 5a), suggesting that *SmTAT1* may be a target gene of SmTGA5. The TAT is the initial enzyme in the tyrosine-derived pathway, and overexpression of *SmTAT1* was able to significantly increase the phenolic acid content in *S. miltiorrhiza* [5]. As a critical enzyme gene in phenolic acid biosynthesis pathway, *SmTAT1* had been shown to be a targeted gene of various TFs, including SmbZIP3 and SmbHLH60 [26, 27]. Indeed, the *as-1* element was found in the *SmTAT1* promoter. EMSA and Y1H showed that SmTGA5 directly binds the *as-1* box in the *SmTAT1* promoter region (Fig. 5b, d). In conclusion, our findings suggest that SmTGA5 has a positive regulatory role in the accumulation of phenolic acid, potentially through the activation of S*mTAT1*. Moreover, the transcript levels of *SmPAL1*, *SmHPPR1*, *SmC4H1*, and *SmCYP98A14* were increased in the T5O lines, whereas SmTGA5 did not activate its expression by Dual-LUC assays, suggesting that SmTGA5 may not directly regulate its expression. In *Artemisia*, AaTGA6 enhances artemisinin content by regulating the transcriptional expression of *AaERF1* [13]. Therefore, there may be some unknown or known TFs in the downstream of SmTGA5 that need to be investigated.

SmNPR4 interacts with SmTGA5, but has an opposite function in phenolic acid accumulation, suggesting that SmNPR4 may influence the function of SmTGA5. We found that SmNPR4 inhibited the transcriptional activation activity of SmTGA5 by Dual-LUC assay (Fig. 6a). Meanwhile, SA treatment abolished the inhibition of SmNPR4 on the transcriptional activation activity of SmTGA5 (Fig. 6b). The interaction between NPR4 and TGAs is not affected by SA [17], indicating that the change in the function of NPR4-TGAs complex may be attributed to a conformational change in NPR4. AtNPR1 enhances the DNA binding activity of AtTGA2 [35]. However, it is unknown whether NPR4, as a paralog of NPR1, affects the activity of TF binding to DNA. SmNPR4 was found to attenuate the DNA binding activity of SmTGA5 by EMSA assay (Fig. 6c). These findings imply that SmNPR4 inhibits phenolic acid biosynthesis by disrupting the activity of SmTGA5 binding to the *SmTAT1* promoter. In the N4T5O lines, *SmTGA5* was found to be highly expressed and *SmNPR4* was found to be poorly expressed, which corresponded to the relatively high expression of the corresponding enzyme genes (Fig. 6). These findings further support the idea that SmNPR4 inhibits the function of SmTGA5.

**Figure 7:**
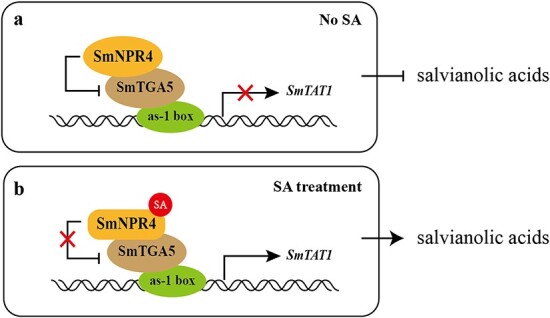
Molecular regulatory network for SA modulating the biosynthesis of salvianolic acid in *Salvia miltiorrhiza*. SmTGA5 directly regulates the salvianolic acid biosynthesis by activating the transcription of *SmTAT1*. The interaction of SmTGA4 and SmTGA5 inhibits the transcriptional activation ability of SmTGA5, and exogenous SA treatment can relieve this inhibition.

Based on the above findings, we propose a working model for the SmNPR4-SmTGA5 module regulating SA-induced phenolic acid biosynthesis in *S. miltiorrhiza* (Fig. 7). The SmTGA5 is a positive regulator of phenolic acid accumulation. In the absence of SA, SmNPR4 repress the transcriptional activation of SmTGA5 on *SmTAT1* gene. After SA treatment, the conformation of SmNPR4 protein bind to SA is changed, which releases its repression of SmTGA5, thereby inducing the transcriptional upregulation of *SmTAT1* and fostering the accumulation of phenolic acids.

## Materials and methods

### Plant materials and SA treatment

Different tissues of 2-year-old wild *S. miltiorrhiza* were acquired from the samples stored in our laboratory [38]. The *S. miltiorrhiza* hairy roots were induced and cultured as previously described [39]. Briefly, 0.3 g FW hairy roots were grown in 50 mL 6,7-V medium and incubated for 21 days at 25°C in a shaker with low shaking speed. Tobacco was grown in the greenhouse at 25°C for use in transient expression analysis approximately 4 weeks later.

SA (Sigma-Aldrich, MO, USA) dissolved in ethanol. The hairy roots culturing after 18 days added SA and same volume of ethanol (control). The final concentration of SA was 50 μM. After treatment for 0, 0.5, 1, 3, 6, 12, 24, 48, and 72 h, the samples were harvested.

Following sample collection, a qRT-PCR assay was performed.

### Bioinformation analysis

The query sequences from *A. thaliana* comprised six *NPR* genes and 10 *TGA* genes (http://www.arabidopsis.org). NPR family sequences and TGA family sequences in *S. miltiorrhiza* genome database were searched by using a Hidden Markov model algorithm (HMMER) (http://www.hmmer.org) [40, 41]. The phylogenetic tree of NPRs and TGAs was constructed using the neighbor-joining method in MEGA v6.0 software [42]. The DNAMAN software was used to perform multiple sequence alignments of protein. Prediction of cis-acting elements in the 2 kb range upstream of enzyme genes by using PlantCARE server (http://bioinformatics.psb.ugent.be/webtools/plantcare/html/), and visualization of promoter element distribution using TBtools package. Different RNA-seq data were obtained and calculated as described previously [38]. The Clustering heat map was created using the FPKMs and visualized using TBtools v1.078 software [43].

### RNA extraction and RT-quantitative PCR

The following is based on the product manuals from Accurate Biology (Hunan, China). The RNA extraction Kit (AG21022) was employed to isolate total RNA from *S. miltiorrhzia* hairy roots. Subsequently, the reverse transcription kit (AG11728) was utilized to perform reverse transcription of RNA into cDNA. Finally, the qPCR kit (AG11701) was applied to conduct qRT-PCR assays on a real-time PCR system (Quant Studio5, ABI, SG). The primers are shown in Table S1 (see online supplementary material). The *Actin* gene was employed as internal control [44]. The expression levels of genes were calculated by the CT method (2^−ΔΔCT^). Three biological replicates were conducted for each treatment in the experiments.

### Subcellular localization analysis

The cDNA of *SmNPR4* and *SmTGA5*, which did not contain termination codon, were cloned into the *Nco*I restriction sites pCsGFPBT vector driven by a 35S promoter through homologous recombination, to construct, respectively, the pCsGFPBT-SmNPR4 and pCsGFPBT-SmTGA5 (primers are shown in Table S1, see online supplementary material). The *N. benthamiana* leaves were infested with *Agrobacterium tumefaciens* GV3101 that transformed recombinant. After 48 h of incubation, detection of GFP fluorescence in tobacco epidermal cells was performed using a confocal laser scanning microscope (Leica, Germany). The pCsGFPBT was chosen as a control in this experiment.

### Plant expression vector construction

For the construction of overexpression and RNAi vector of *SmNPR4*, the ORF of *SmNPR4*, as well as the sequence containing partial 3′-translated region and 3′-untranslated regions, were subcloned into pDONR207, and then cloned into the pK7WG2R and pK7GWIWG2R using Gateway technology (Invitrogen, USA), respectively [45]. The ORF of *SmTGA5* was amplified in sense and antisense orientations, and then inserted into the restriction sites *Hind*III and *BstE*II of the pCAMBIA1304 through homologous recombination. The sequence containing the CaMV35S promoter, the full-length ORF of SmTGA5 and terminator were amplified from pCAMBIA1304-SmTGA5 and inserted into the restriction sites *Xho*I and *Kpn*I of the pK7WG2R-SmNPR4 vector to obtain a double genes overexpression vector (Fig. S9a, see online supplementary material).

### Plant transformation and transformation selection

Those recombinants were transfected into *S. miltiorrhzia* via the *Agrobacterium rhizogenes* ATCC15834 to produce transgenic hairy roots, and all transformation steps as previously described [46]. Two strategies had been applied to confirm the positive transgenic lines. First, transgenic lines were subjected to genomic DNA extraction, and then PCR amplified with gene-specific primers (listed in Table S1, see online supplementary material) to identify positive lines at the DNA levels. Finally, the transcript levels of target genes (*SmNPR4* and/or *SmTGA5*) in transgenic lines were measured by qRT-PCR at the RNA level.

### Extraction and detection of phenolic acids

The methods for extracting phenolic acids from transgenic lines (N4O, N4R, T5O, T5A, and N4T5O) and controls were according to a previous report [46]. Briefly, the hairy roots were synchronized cultured for 21 days, followed by drying at 45°C and grinding into a dry powder. Subsequently, 20 mg powder was added with 4 mL 70% methanol, soaked in the dark for 8 h, sonicated for 45 min. After centrifugation, the supernatant was filtered using the filter (0.22 μm, Millipore, US) stored at 4°C.

Two methods were used to detect the extracts. In HPLC assays, the detection methods of RA and Sal B content were carried out as previously reported [46]. According to a modified Folin–Ciocalteu method, TPA were detected, and the specific operation steps were as described above [47].

### Yeast two-hybrid (Y2H) and yeast one-hybrid (Y1H) assays

In the Y2H assay, we cloned the ORFs of *SmTGA5* and *SmNPR4* into the pGBKT7 and the pGADT7 vector, respectively (primers are shown in Table S1, see online supplementary material). The yeast Y2H, co-transformed with pGBKT7-SmTGA5 and pGADT7-SmNPR4, was treated as the experimental group. As a negative control, the empty vector and other recombined vector (pGBKT7-SmTGA5 and pGADT7; pGADT7-SmNPR4 and pGBKT7) were also co-transformed. After selection of monoclonal strains on DDO solid medium, their growth was assessed on QDO/X solid medium. The yeast strains were incubated under 29°C incubator for 3–4 days and their growth was observed.

In the Y1H assay, we generated the pTAT1-AbAi by cloning the *SmTAT1* promoter sequence containing the *as-1* box (TGACG) into the pAbAi vector. The pGADT7-SmTGA5 was generated by cloning the ORF of *SmTGA5* into the pGADT7 vector. The Y1H assays were performed based on the product manuals (Clontech, CA, USA).

### Firefly luciferase complementation imaging (LCI) assay

The LCI assays were based on those previously described [48]. In short, we created nLUC-SmNPR4 and cLUC-SmTAG5 recombinants, and transformed into GV3101. Then, the positive strains were injected into 34-day-old *N. benthamiana* leaves. Two days after incubation, Beetle Luciferin (50 mM) was applied to the leaves and left in the dark for 5 minutes before fluorescence observation was performed. The LCI images were acquired with an In Vivo Imaging System (Lumazone Pylon 2048B, Princeton, USA). This experiment was repeated 5–8 times.

### Pull-down assay

The ORF of *SmNPR4* and *SmTGA5* without termination codon was amplified and cloned into pGEX-4 T-1 vector including GST-tag and pET32a vector including His-tag, respectively (primers are shown in Table S1, see online supplementary material). GST protein and GST-SmNPR4 fusion protein were using glutathione sepharose beads, while His protein and His-SmTGA5 fusion protein were purified using Ni-NTA agarose beads (Sangon, Shanghai, China). The purification of recombinant proteins and tag protein and pull-down assays were performed according to the previous reports [49].

### Dual-luciferase (dual-LUC) assay

The promoters of *SmHPPR1*, *SmPAL1*, *SmC4H1*, *SmRAS1*, *SmCYP98A14,* and *SmTAT1*genes as well as mutant promoter of *SmTAT1* gene were amplified and fused to the pGreenII 0800-LUC vector, respectively (primers are shown in Table S1, see online supplementary material). The CaMV35S promoter-controlled renilla luciferase (Rluc) was employed as the endogenous control. The effector plasmids were pCsGFPBT-SmNPR4 and pCsGFPBT-SmTGA5, and the control plasmid was pCsGFPBT. Dual-LUC assays were performed according to the previous reports [50].

### Electrophoretic mobility shift assay (EMSA)

All probes (biotin-labeled, unlabeled and mutated probes) were synthesized by Sangon Biotech (Shanghai, China). The EMSA assay was performed as per the supplier’s instructions (GS009, Beyotime, Shanghai, China). In the experiment, fusion proteins were mixed with a biotin-labeled probe, while tag protein was mixed with a biotin-labeled probe and fusion proteins mixed with a mutated biotin-labeled probe served as negative controls. Unlabeled probes were used in competition assays with a molar ratio of 1:10; and 1:100. All the mixtures were incubated at 20 to 25°C for 20 minutes, and then separated into free and bound probes using acrylamide gel electrophoresis.

## Acknowledgements

We are grateful to the Molecular Platform at Northwest A&F University’s College of Life Sciences. Thanks to YongFeng Xie from Weinan Normal University (Shaanxi, China) for her help with the instruction of experiment. The research was financially supported by the National Natural Science Foundation of China (31670301, 32270278), the Natural Science Foundation of Shaanxi Province (2022JM-099), and the Innovation Training Program for College Students (202210712222).

## Author contributions

J.D. and P.M. designed the research. M.D., S.Z., R.H., and Y.X. performed the experiments. M.D. analysed the data and wrote the manuscript. B.Z. revised the paper.

## Data availability

The data that support the results of this paper are available in this paper and its supplementary materials.

## Conflict of interest

 The authors declare that they have no conflict of interests.

## Supplementary data

Supplementary data is available at *Horticulture Research* online.

## Supplementary Material

Web_Material_uhad066
