# Supplementary material for: The SmNPR4-SmTGA5 module regulates SA-mediated phenolic acid biosynthesis in Salvia miltiorrhiza hairy roots
Source: Hortic Res. 2023 Apr 10;10(5):uhad066. doi: 10.1093/hr/uhad066 (PMC10208894; doi:10.1093/hr/uhad066)
Supplement: Web_Material_uhad066 [file web_material_uhad066.zip › Supplementary materials.docx]

**The SmNPR4-SmTGA5 module regulates SA-mediated** **phenolic acid biosynthesis in** ***Salvia miltiorrhiza* hairy roots**

Meiling Ding^1^, Bin Zhang^1^, Shuo Zhang^1^, RongRong Hao^1^, Yu Xia^1^, Pengda Ma^1^,^＊^ and Juane Dong^1^,^＊^

^1^ College of Life Sciences, Northwest A & F University, Yangling, China

^＊^ Correspondence: Pengda Ma (e-mail: mapengda@163.com, Tel: +86 029 87092262) and Juane Dong (e-mail: dje009@126.com, Tel: +86 029 87092904)

**Fig. S1** Protein sequence Alignment of SmNPR4 with AtNPR3/4. BTB/POZ, ankyrin repeats domain, NPR1_like_C domain and putative EAR motif (VDLNETP) are underlined in red. The asterisk represents the residue of the SA binding.

**Fig. S2** PCR screening of *SmNPR4* overexpressing or RNAi lines.

**Fig. S3** Cis-acting elements for enzyme gene promoters in *S. miltiorrhiza*. Cis-acting elements were divided into 3 classes, environmental stress-related elements, hormone responsive elements, and development-related elements, which are represented by the up triangular, round rectangle, and down triangular shapes, respectively.

**Fig. S4** Heatmap of *SmTGAs* and enzyme genes expression profiles at different treatment.

**Fig. S5** Subcellular localization analysis of SmTGA5 in tobacco leaves.

**Fig. S6** Expression pattern analysis of *SmTGA5*. (a) Tissue-specific expression of *SmTGA5*. The expression levels were normalized to values from roots. (b) Relative expression profile of the *SmTGA5* in the 50 μM SA-treated hairy roots. The expression levels were normalized to values from 0 h. Bars are means ±SD from three independent biological replicates were using he Student's t-test (**P < 0.01, *P < 0.05).

**Fig. S7** PCR Screening of *SmTGA5* overexpressing or anti-sense lines.

**Fig. S8** Overexpression of SmTGA5 significantly activates the *SmTAT1* promoter. A mutated version (*Mut-proSmTAT1*) was designed as a negative control. All data represent the mean ± SD of three independent experiments. Statistical significance was assessed with Student’s t-test (**P < 0.01; *P < 0.05).

**Fig. S9** Schematic diagram of vector construction and PCR screening of dual-overexpressing lines. (a) Schematic representation of the pCAMBIA1304-SmTGA5, pK7WG2R-SmNPR4 and pK7WG2R-SmNPR4-SmTGA5 constructs. (b) PCR screening of dual-overexpressing lines.

**Table S1** Primers employed for qRT-PCR, positive hairy roots selection and vector construction.

**Table S2** The genes implicated in this paper.


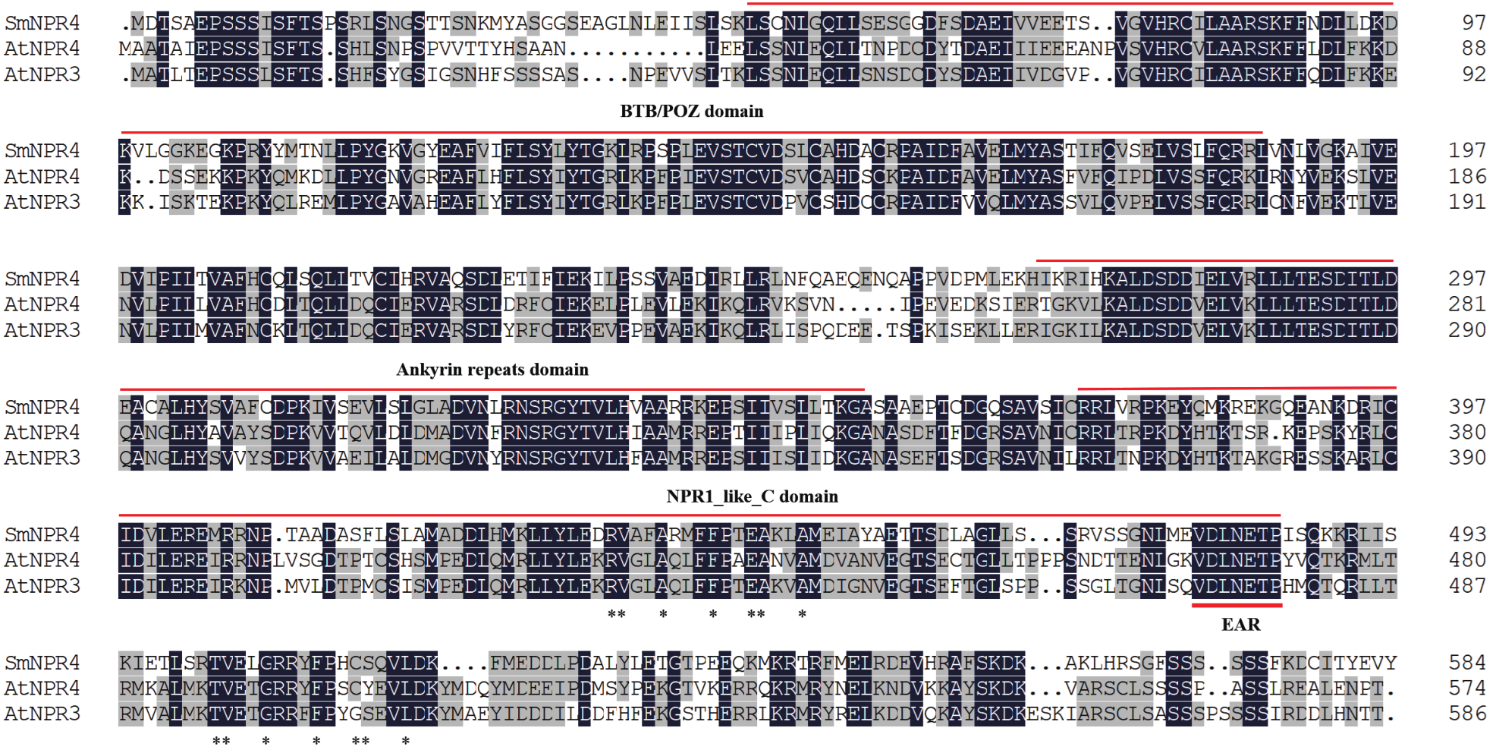
**Fig. S1**


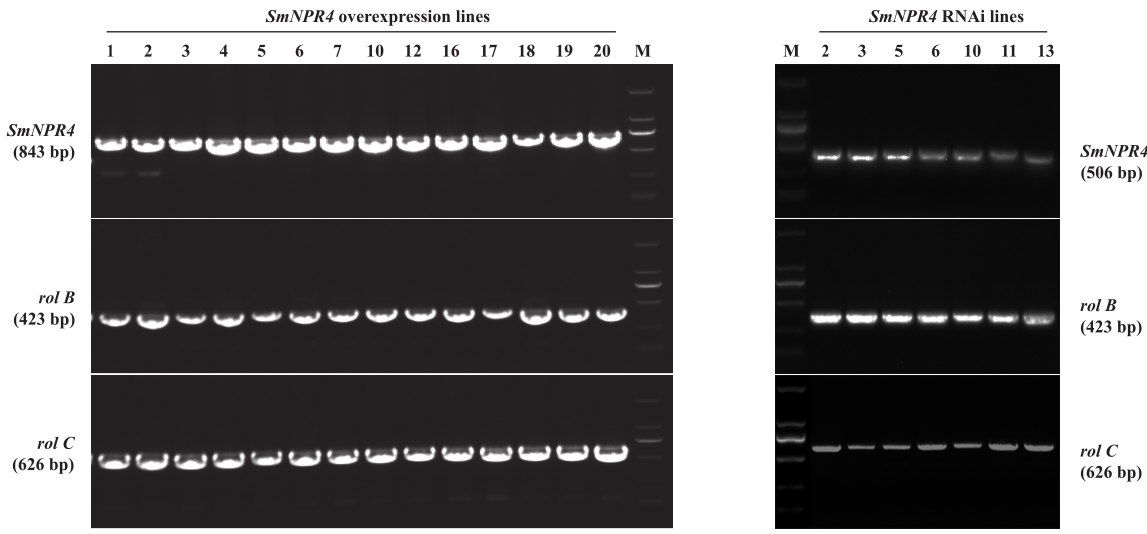
**Fig. S2**


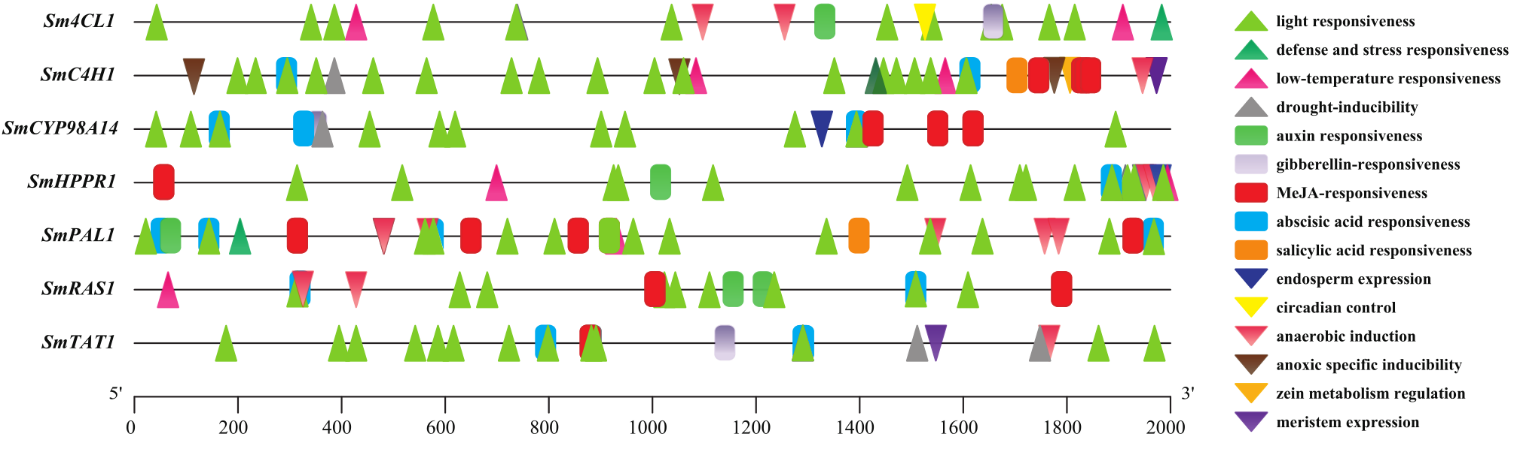
**Fig. S3**


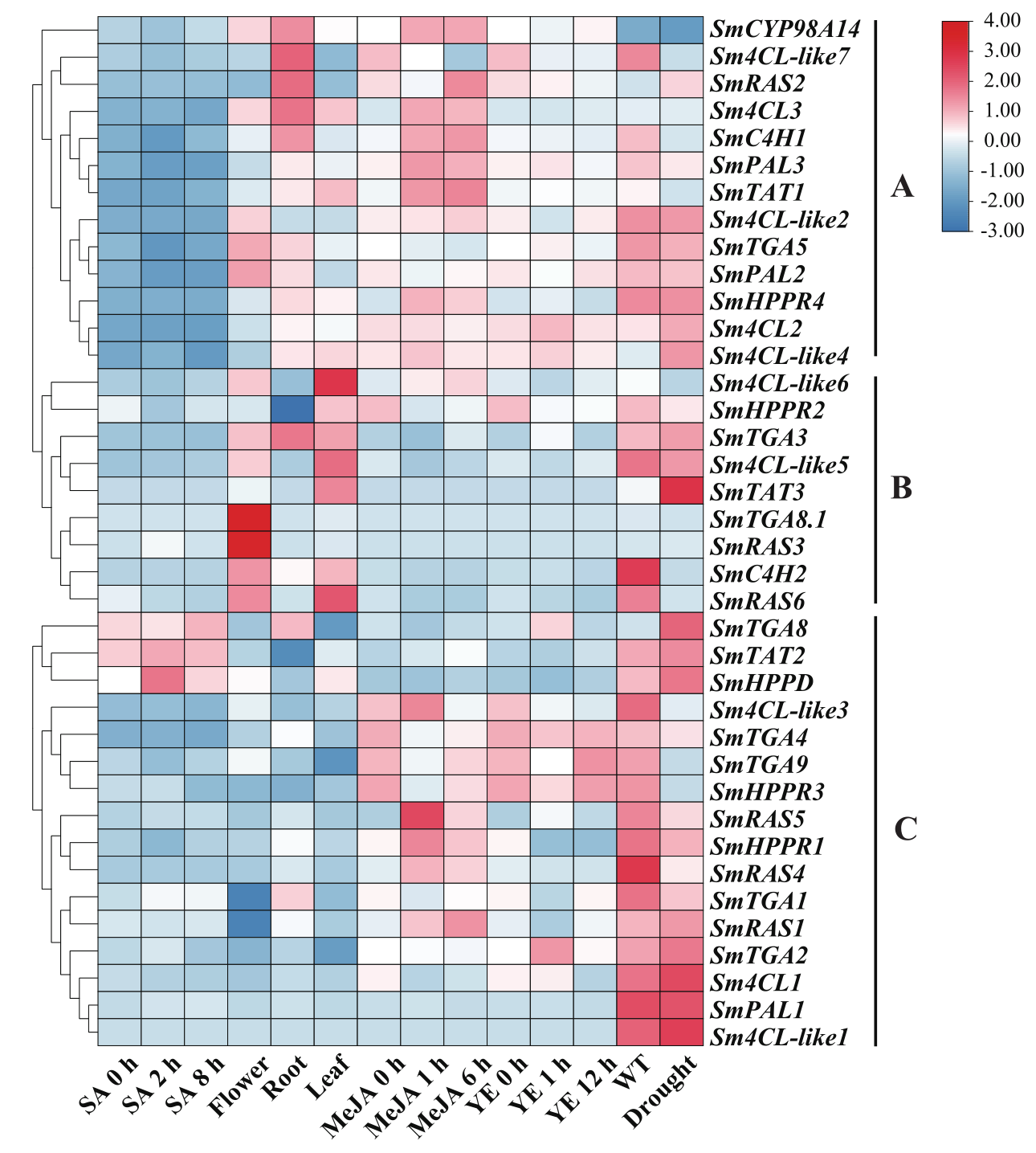


**Fig. S4**


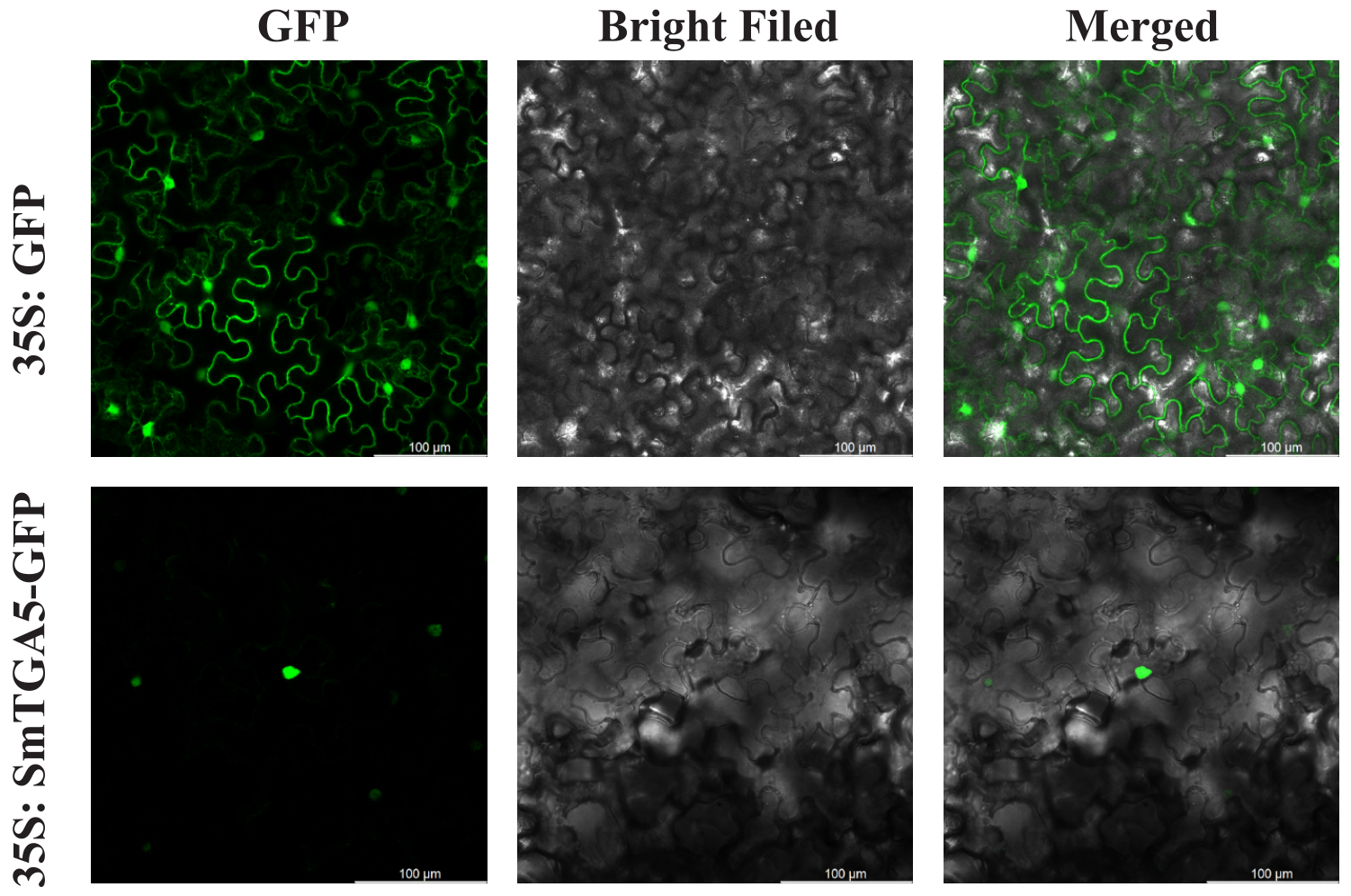


**Fig. S5**


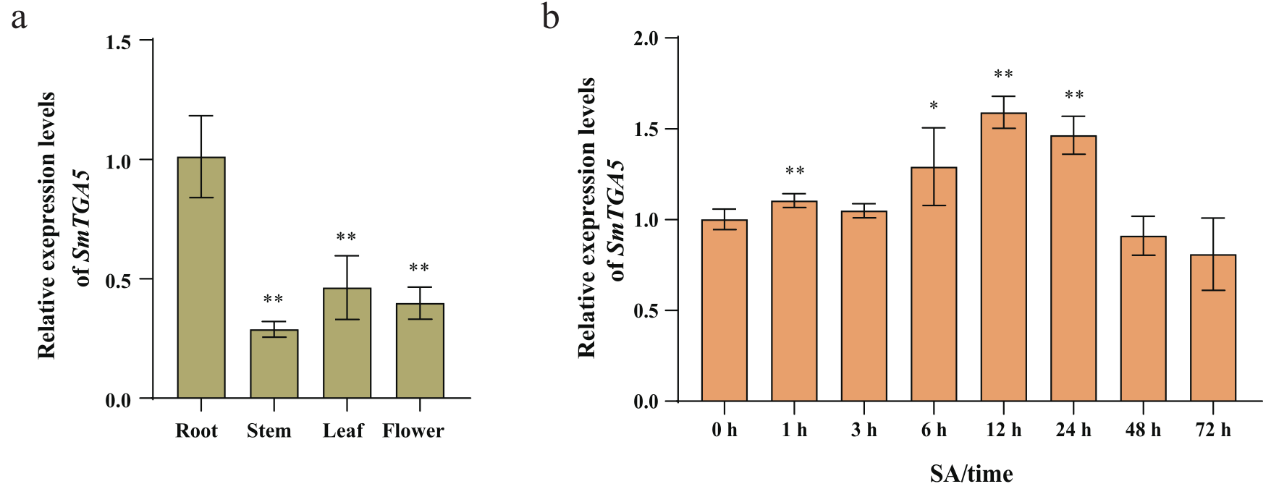
**Fig. S6**

**Fig. S7**


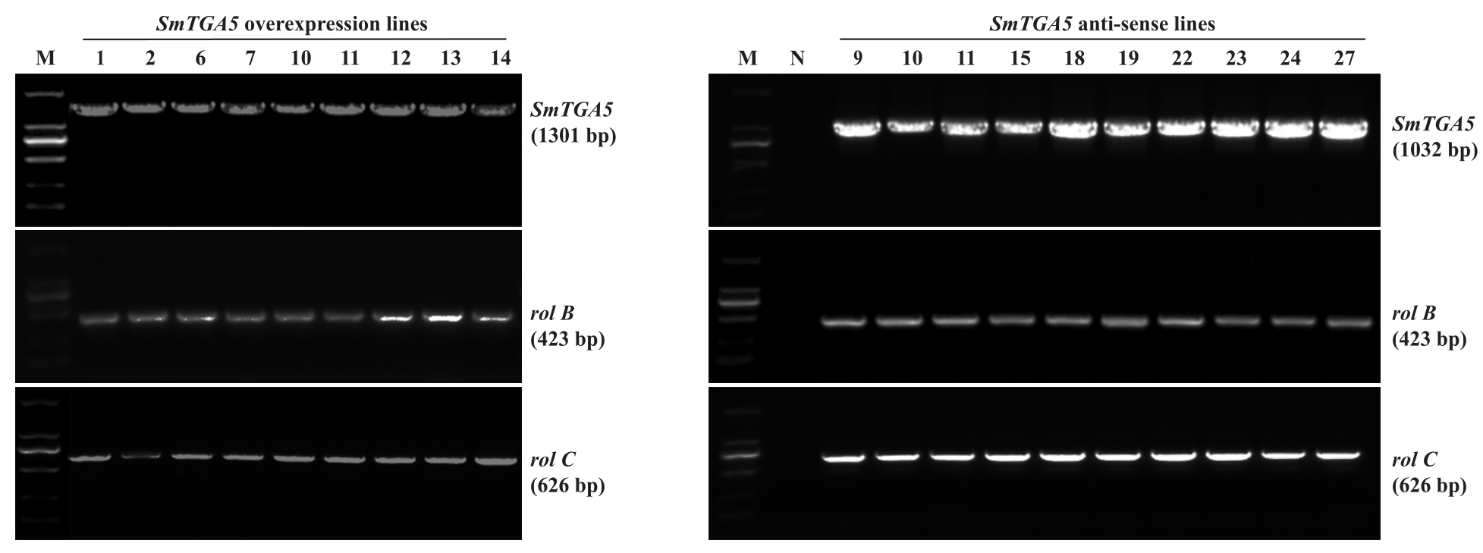


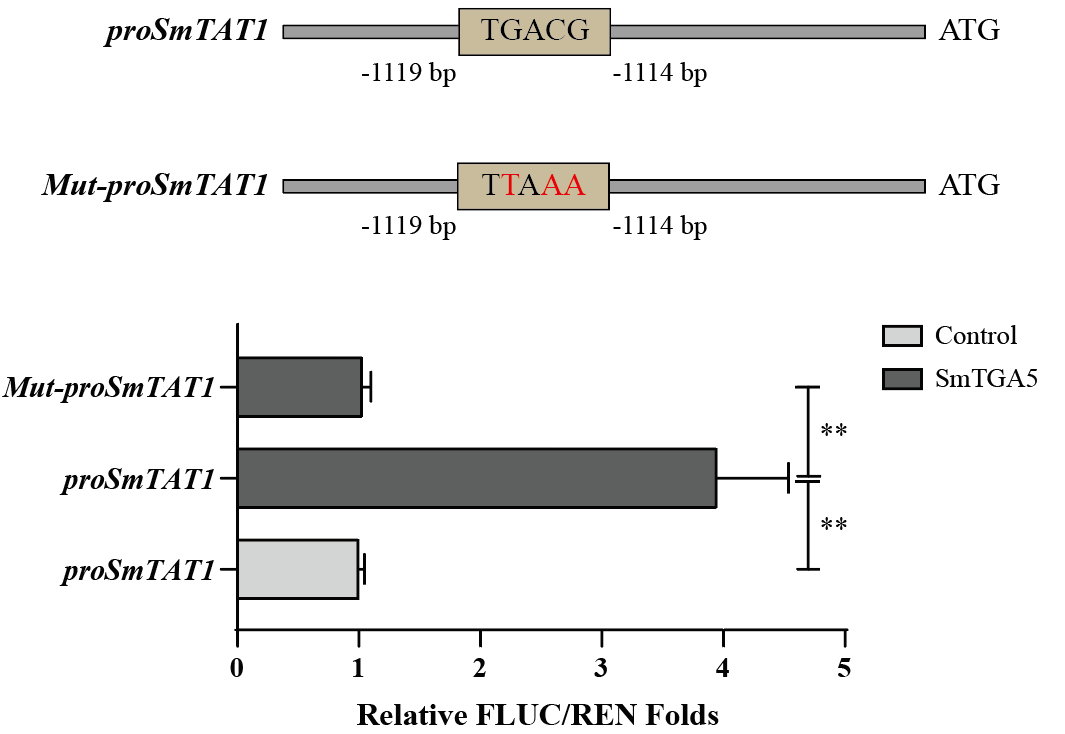
**Fig. S8**

**
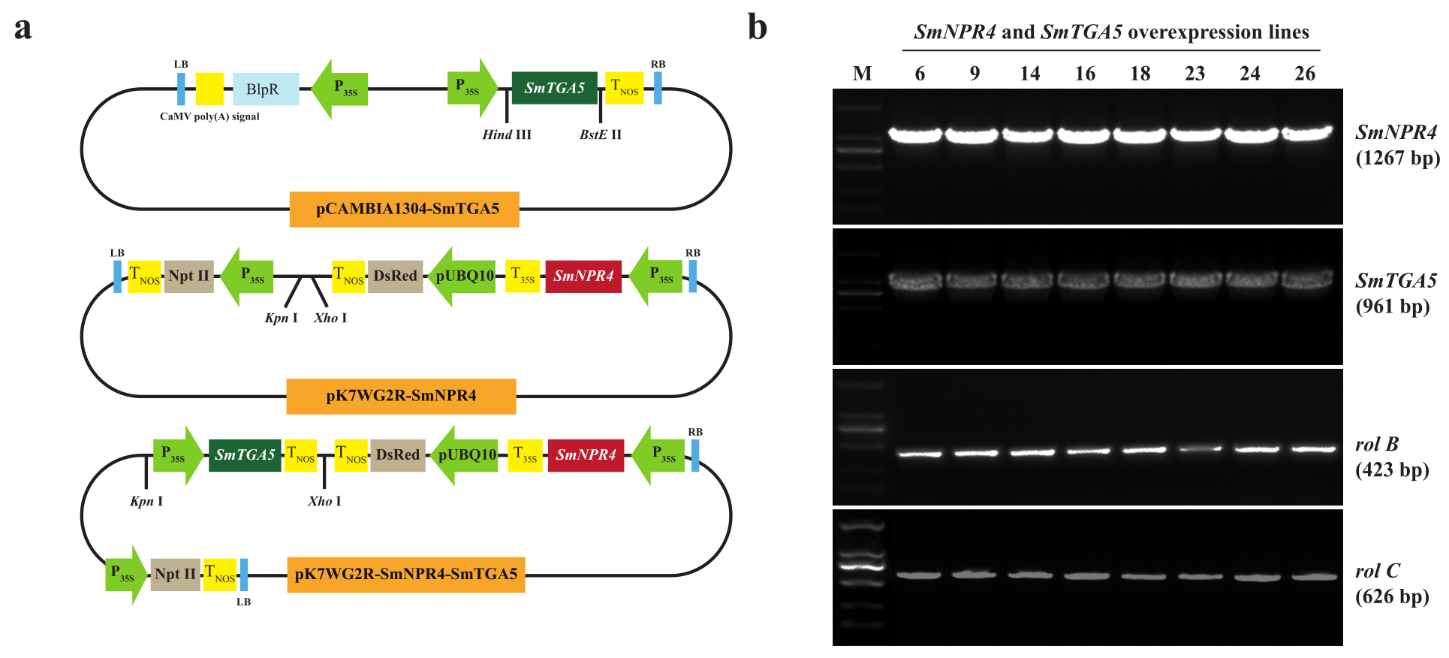
Fig. S9**

**Table S1 Primers employed for qRT-PCR, positive hairy roots selection and vector construction**

| **Assays** | **Primer Name** | **Primer sequence (5'-3')** |
| --- | --- | --- |
| Gene Clone | SmNPR4-F | ATGGATACTTCTGCAGAGCC |
|  | SmNPR4-R | TCAGATTTTGTATACTTCGTAAGTTATG |
|  | SmTGA5-F | ATGGCTAGAGCAACTGTAAATATTG |
|  | SmTGA5-R | TTAGCCTTCTTTGGGCCTTG |
| Transgenic lines | pK7WG2R-SmNPR4-F | GGGGACAAGTTTGTACAAAAAAGCAGGCTTAATGGATACTTCTGCAGAGCC |
|  | pK7WG2R-SmNPR4-R | GGGGACCACTTTGTACAAGAAAGCTGGGTATCAGATTTTGTATACTTCGTAAGTTATG |
|  | pK7GWIWG2_II-SmNPR4-F | GGGGACAAGTTTGTACAAAAAAGCAGGCTTAGGTTCACAGAGCATTCAACAAGG |
|  | pK7GWIWG2_II-SmNPR4-R | GGGGACCACTTTGTACAAGAAAGCTGGGTAGCAGCATCCAAAAAGATTTATTACA |
|  | pCAMBIA1304-SmTGA5-F | AACACGGGGGACTTCTAGAAAGCTATGGCTAGAGCAACTGTAAATATTG |
|  | pCAMBIA1304-SmTGA5-R | GGAAATTCGAGCTGGTCACCTTAGCCTTCTTTGGGCCTTG |
|  | pCAMBIA1304-antiSmTGA5-F | AACACGGGGGACTTCTAGAAAGCTTTAGCCTTCTTTGGGCCTTG |
|  | pCAMBIA1304-antiSmTGA5-R | GGAAATTCGAGCTGGTCACCATGGCTAGAGCAACTGTAAATATTG |
|  | N4T5-F | TCACACTGGCGGCCGCTCGAGAATATCAAAGATACAGTCTCAGAAGACCAAAG |
|  | N4T5-R | TGTATGATAATTCGCGGTACCGATCTAGTAACATAGATGACACCGCGC |
| Y2H | pGBKT7-SmTGA5-F | ATGGCCATGGAGGCCGAATTCATGGCTAGAGCAACTGTAAATATTG |
|  | pGBKT7-SmTGA5-R | ATGCGGCCGCTGCAGGTCGACTTAGCCTTCTTTGGGCCTTG |
|  | pGADT7-SmNPR1-F | GGAGGCCAGTGAATTCATGGACGGCCGAACCGTT |
|  | pGADT7-SmNPR1-R | CACCCGGGTGGAATTCTTAGTTTTGGGACGGGAGCG |
|  | pGADT7-SmNPR4-F | GGAGGCCAGTGAATTCATGGATACTTCTGCAGAGCC |
|  | pGADT7-SmNPR4-R | CACCCGGGTGGAATTCTCAGATTTTGTATACTTCGTAAGTTATG |
| Y1H | pGADT7-SmTGA5-F | GGAGGCCAGTGAATTCATGGCTAGAGCAACTGTAAATATTG |
|  | pGADT7-SmTGA5-R | CACCCGGGTGGAATTCTTAGCCTTCTTTGGGCCTTG |
|  | pABAi-pTAT1-F | AAAAGCTTGAATTCGAGCTCTAAATCTCCGCTATTGTTCACATT |
|  | pABAi-pTAT1-R | AGCACATGCCTCGAGGTCGACACTCAATTTTGAAATGGTTCTCGT |
| LCI | nLUC-SmNPR4-F | CACGGGGGACGAGCTCGGTACCATGGATACTTCTGCAGAGCCA |
|  | nLUC-SmNPR4-R | ACGCGTACGAGATCTGGTCGACGATTTTGTATACTTCGTAAGTTATGCA |
|  | cLUC-SmTGA5-F | CACGGGGGACGAGCTCGGTACCATGGCTAGAGCAACTGTAAATATTG |
|  | cLUC-SmTGA5-R | ACGCGTACGAGATCTGGTCGACGCCTTCTTTGGGCCTTGC |
| Dual-LUC | pGreenⅡ-0800-luc-ProPAL1-F | GTACCGGGCCCCCCCTCGAGGTCGACAGCCATTTATTATTTCCTTCCTCTTT |
|  | pGreenⅡ-0800-luc-ProPAL1-R | GGCTGCAGGAATTCGATATCAAGCTTTTAGTGGCAAAACAAGGACGAGA |
|  | pGreenⅡ-0800-luc-ProC4H1-F | GTACCGGGCCCCCCCTCGAGGTCGACAATTGTAAATACTAAATACCCAGG |
|  | pGreenⅡ-0800-luc-ProC4H1-R | GGCTGCAGGAATTCGATATCAAGCTTTTTAAAATGGTTTGCGGTC |
|  | pGreenⅡ-0800-luc-ProTAT1-F | GTACCGGGCCCCCCCTCGAGGTCGACACGAACATTCATGATTACCCAAA |
|  | pGreenⅡ-0800-luc-ProTAT1-R | GGCTGCAGGAATTCGATATCAAGCTTGAAACATAGAGCATTCACACGGA |
|  | pGreenⅡ-0800-luc-ProHPPR1-F | GTACCGGGCCCCCCCTCGAGGTCGACATTTTTACGAAGTGAAAGAAATA |
|  | pGreenⅡ-0800-luc-ProHPPR1-R | GGCTGCAGGAATTCGATATCAAGCTTTTCTACTATTTTATATTGTTTCAATA |
|  | pGreenⅡ-0800-luc-ProRAS1-F | GTACCGGGCCCCCCCTCGAGGTCGACTAGCATCGGTAACATGAGCAAAG |
|  | pGreenⅡ-0800-luc-ProRAS1-R | GGCTGCAGGAATTCGATATCAAGCTTAATAAGGTCTGGTATAATACACCGC |
|  | pGreenⅡ-0800-luc-ProCYP98A14-F | GTACCGGGCCCCCCCTCGAGGTCGACCTTTTTCTTAAAATTCGTGACACTCC |
|  | pGreenⅡ-0800-luc-ProCYP98A14-R | GGCTGCAGGAATTCGATATCAAGCTTGGTGGTGAAGGGTAGTTGGC |
|  | pGreenⅡ-0800-luc -ProTAT1-mut-F1 | GTACCGGGCCCCCCCTCGAGGTCGACACGAACATTCATGATTACCC |
|  | pGreenⅡ-0800-luc-ProTAT1-mut-R1 | GCAGTCGTGATACATTTAACTT |
|  | pGreenⅡ-0800-luc-ProTAT1-mut-F2 | TTACTTTAAAGTTAAATGTATCACG |
|  | pGreenⅡ-0800-luc-ProTAT1-mut-R2 | GGCTGCAGGAATTCGATATCAAGCTTGAAACATAGAGCATTCACACG |
|  | pCsGFPBT-SmNPR4-F | TCACCATTTACGAACGATAGCCATGATGGATACTTCTGCAGAGCCA |
|  | pCsGFPBT-SmNPR4-R | GCTCCTCGCCCTTGCTCACCATGGCGATTTTGTATACTTCGTAAGTTATGCA |
|  | pCsGFPBT-SmTGA5-F | TCACCATTTACGAACGATAGCCATGATGGCTAGAGCAACTGTAAATATTG |
|  | pCsGFPBT-SmTGA5-R | GCTCCTCGCCCTTGCTCACCATGGCGCCTTCTTTGGGCCTTGC |
| qRT-PCR | q-SmACT-F | GGTGCCCTGAGGTCCTGTT |
|  | q-SmACT-R | AGGAACCACCGATCCAGACA |
|  | q-SmPAL1-F | GTGAAGAACACCGTGAGCCAG |
|  | q-SmPAL1-R | GTCGTCGA TGTAGGCGAAAAC |
|  | q-SmC4H1-F | TCTTGCGTTGCCTA TTCT |
|  | q-SmC4H1-R | CAA TGGTCGAGTGCTTCAA |
|  | q-Sm4CL1-F | ATTCGCATTCGCATTTCTCGG |
|  | q-Sm4CL1-R | GCGGCGTAGTGCTTCACCTTT |
|  | q-SmTAT1-F | AGTAGACGTGCCTGCTCT |
|  | q-SmTAT1-R | TGGCTATCCAACTCCTTC |
|  | q-SmHPPR1-F | CCTGACTCCAGAAACAACCCAC |
|  | q-SmHPPR1-R | ACCCAGACGACCCTCCACA |
|  | q-SmRAS1-F | CAGTTTCCGGTGCCCTAA T |
|  | q-SmRAS1-R | TGATGGCGACGAACAAGC |
|  | q-SmCYP98A14-F | CCTCAACGTCGTCGTTTCCA |
|  | q-SmCYP98A14-R | AGTCCGCCCAAA TCAAA TCC |
| Pull-down | pGEX4T1-SmNPR4-F | TGGATCCCCGGAATTCATGGATACTTCTGCAGAGCC |
|  | pGEX4T1-SmNPR4-R | GTCGACCCGGGAATTCTCAGATTTTGTATACTTCGTAAGTTATG |
|  | pET32a-SmTGA5-F | CCATGGCTGATATCGGATCCGAATTCATGGCTAGAGCAACTGTAAATATTG |
|  | pET32a-SmTGA5-R | AAGCTTGTCGACGGAGCTCGAATTCGCCTTCTTTGGGCCTTGC |
| EMSA | Probe-pTAT1-F | TTTAAAGTGACGTGTATCATTTAAAGTGACGTGTATCATTTAAAGTGACGTGTATCA |
|  | Probe-pTAT1-R | TGATACACGTCACTTTAAATGATACACGTCACTTTAAATGATACACGTCACTTTAAA |
|  | Mut-Probe-pTAT1-F | TTTAAAGTTAAATGTATCATTTAAAGTTAAATGTATCATTTAAAGTTAAATGTATCA |
|  | Mut-Probe-pTAT1-R | TGATACATTTAACTTTAAATGATACATTTAACTTTAAATGATACATTTAACTTTAAA |
| positive hairy lines selection | rolB-F | GCTCTTGCAGTGCTAGATTT |
|  | rolB-R | GAAGGTGCAAGCTACCTCTC |
|  | rolC-F | CTCCTGACATCAAACTCGTC |
|  | rolC-R | TGCTTCGAGTTATGGGTACA |
|  | TGA5-OE-F | GAGCACGACACACTTGTCTACTC |
|  | TGA5-OE-R | TCGTTGAAGTGTGTCGCAGTG |
|  | TGA5-anti-F | GAGCACGACACACTTGTCTACTC |
|  | TGA5-anti-R | ACGCTGAAAGGCAACGCA |
|  | NPR4-OE-F | GACGCACAATCCCACTATCC |
|  | NPR4-OE-R | CAAGCTCCGAGACCTGAA |
|  | NPR4-RNAi-F | GACGCACAATCCCACTATCC |
|  | NPR4-RNAi-R | GCAGCATCCAAAAAGATTTATTACA |
|  | N4T5-NPR4-F | GACGCACAATCCCACTATCC |
|  | N4T5-NPR4-R | ACCTAGACTAAGCACCTCCG |
|  | N4T5-TGA5-F | GACGCACAATCCCACTATCC |
|  | N4T5-TGA5-F | TCGTTGAAGTGTGTCGCAGTG |

**Table S2 The genes implicated in this paper**

| Gene Name | Gene ID | Orthologous |
| --- | --- | --- |
| SmNPR1 | c33824.graph_c0 | AtNPR1 |
| SmNPR3 | c35608.graph_c0 | - |
| SmNPR4 | c16843.graph_c0 | AtNPR4 |
| SmNPR5 | SMil_00007973 | AtNPR5 |
| SmNPR6 | SMil_00016435 | - |
| SmTGA1 | SMil_00005206 | AtTGA1 |
| SmTGA4 | SMil_00018706 | - |
| SmTGA2 | SMil_00000233 | AtTGA2 |
| SmTGA5 | SMil_00004488 | - |
| SmTGA3 | SMil_00007088 | AtTGA3 |
| SmTGA8 | SMil_00007005 | AtTAG8 |
| SmTGA8.1 | SMil_00026032 | - |
| SmTGA9 | SMil_00015267 | AtTGA9 |
