# Supplementary figures and images for: The SmNPR4-SmTGA5 module regulates SA-mediated phenolic acid biosynthesis in Salvia miltiorrhiza hairy roots
Source: Hortic Res. 2023 Apr 10;10(5):uhad066. doi: 10.1093/hr/uhad066 (PMC10208894; doi:10.1093/hr/uhad066)

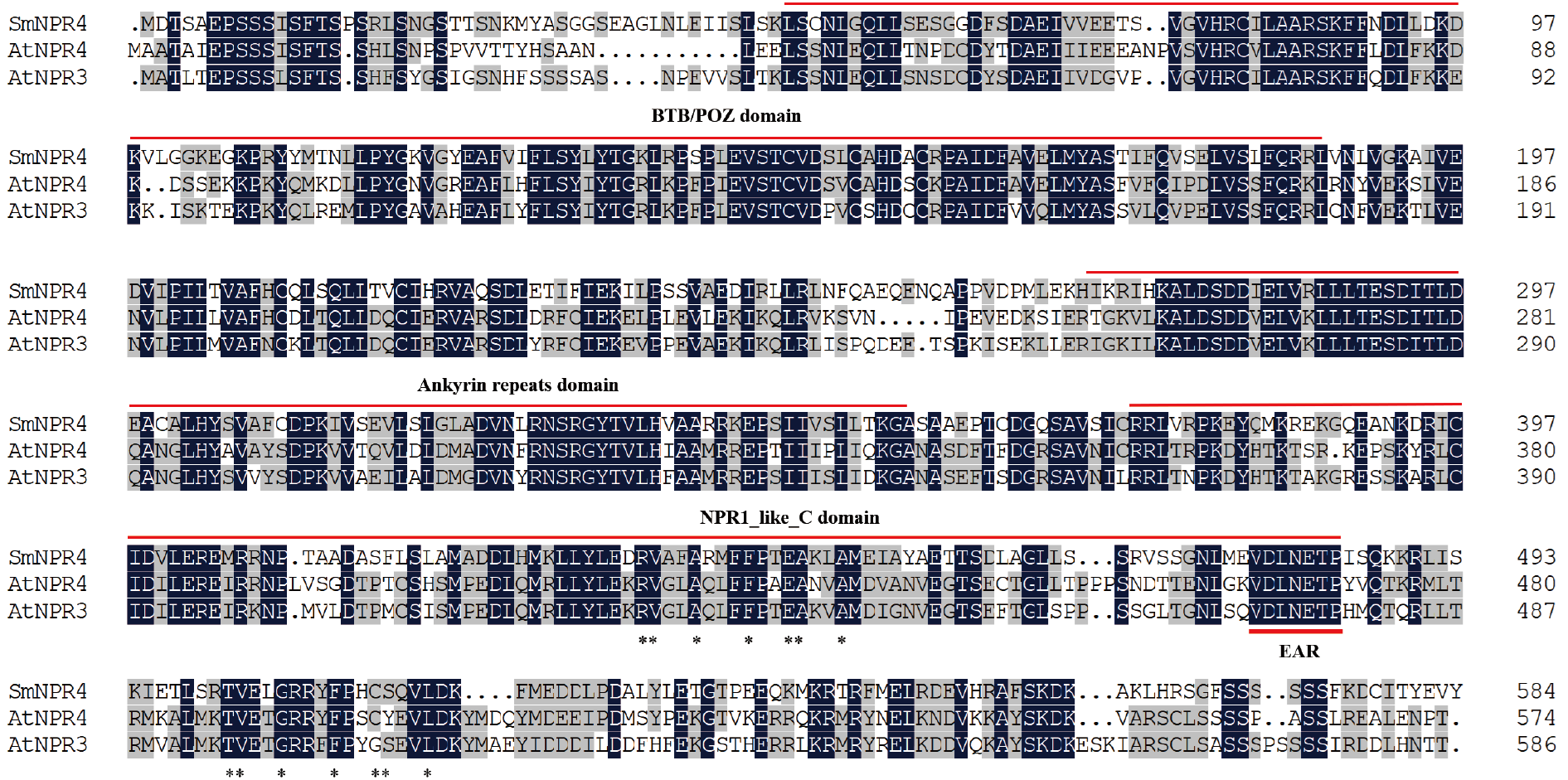

Supplement: Web_Material_uhad066 [file web_material_uhad066.zip › Fig. S1.tif]

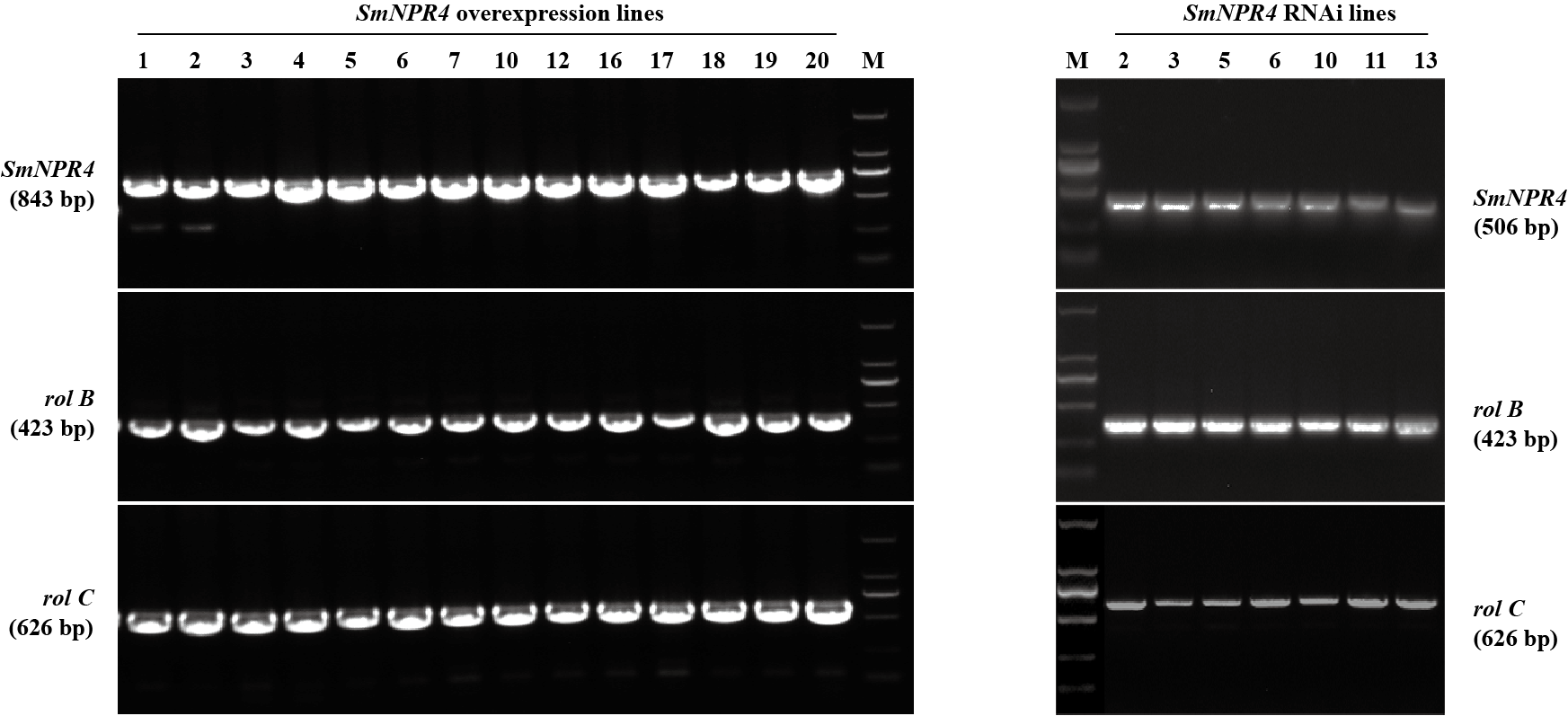

Supplement: Web_Material_uhad066 [file web_material_uhad066.zip › Fig. S2.tif]

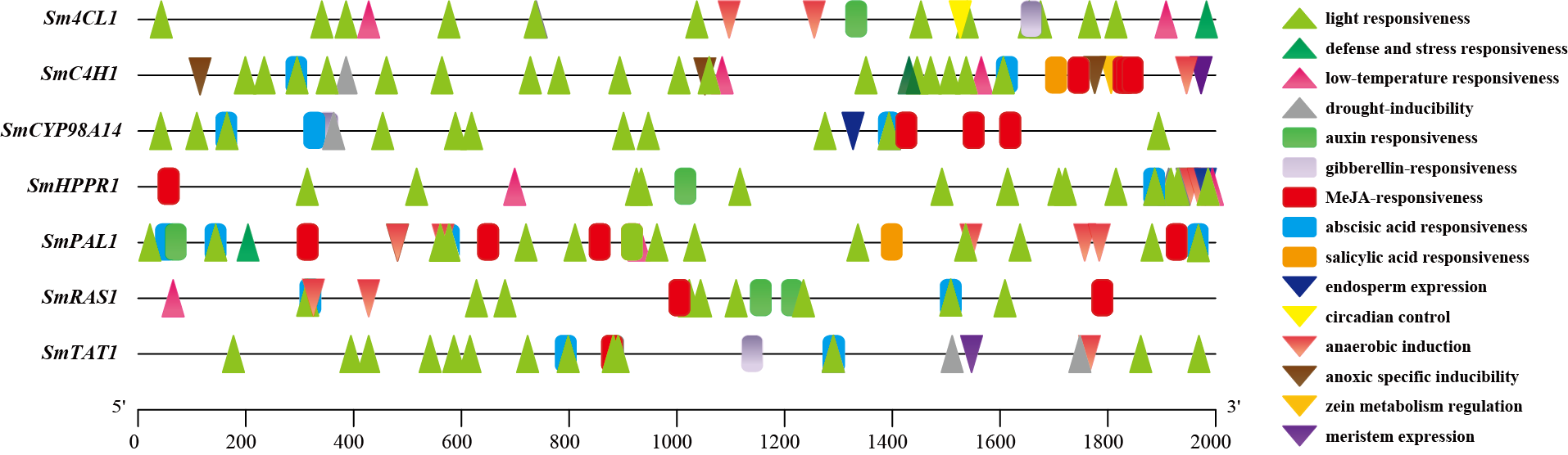

Supplement: Web_Material_uhad066 [file web_material_uhad066.zip › Fig. S3.tif]

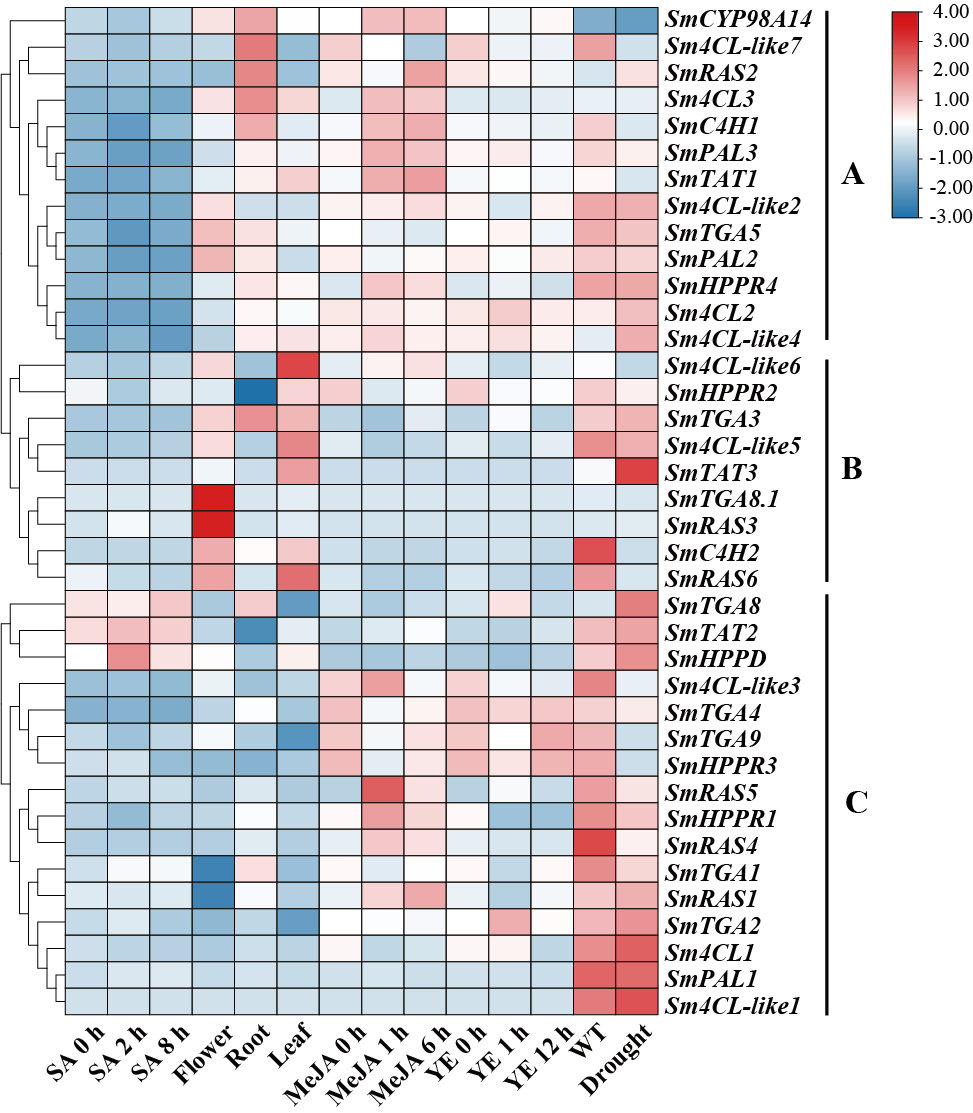

Supplement: Web_Material_uhad066 [file web_material_uhad066.zip › Fig. S4.tif]

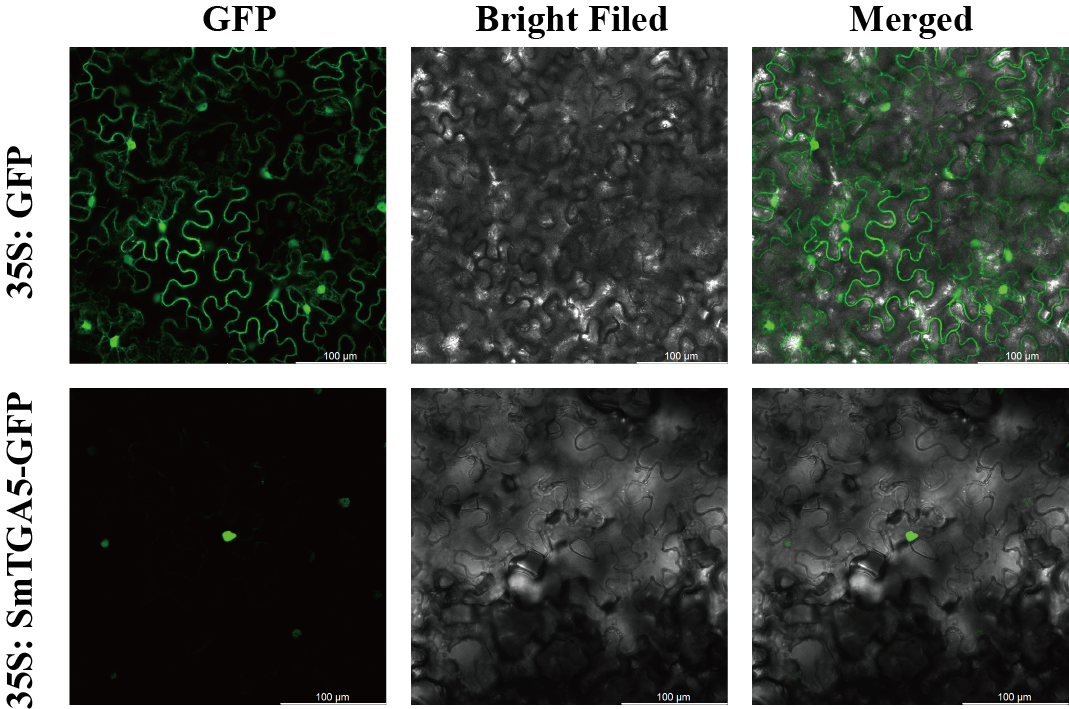

Supplement: Web_Material_uhad066 [file web_material_uhad066.zip › Fig. S5.tif]

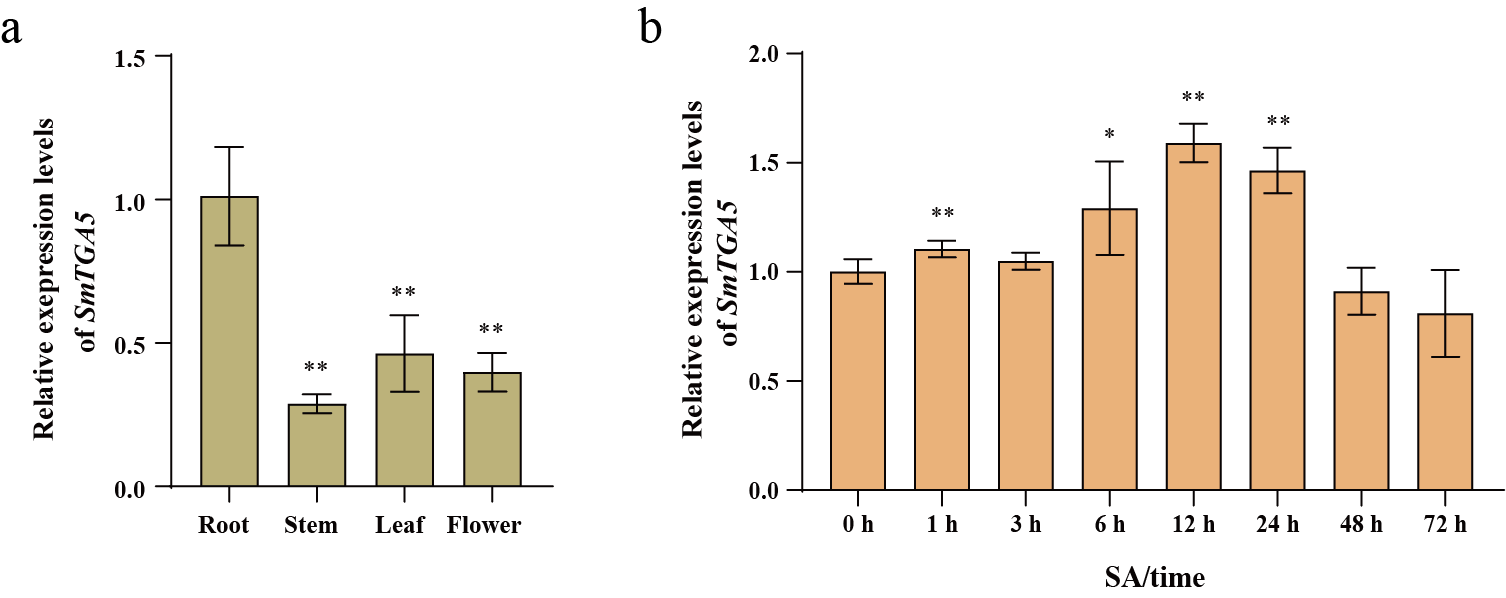

Supplement: Web_Material_uhad066 [file web_material_uhad066.zip › Fig. S6.tif]

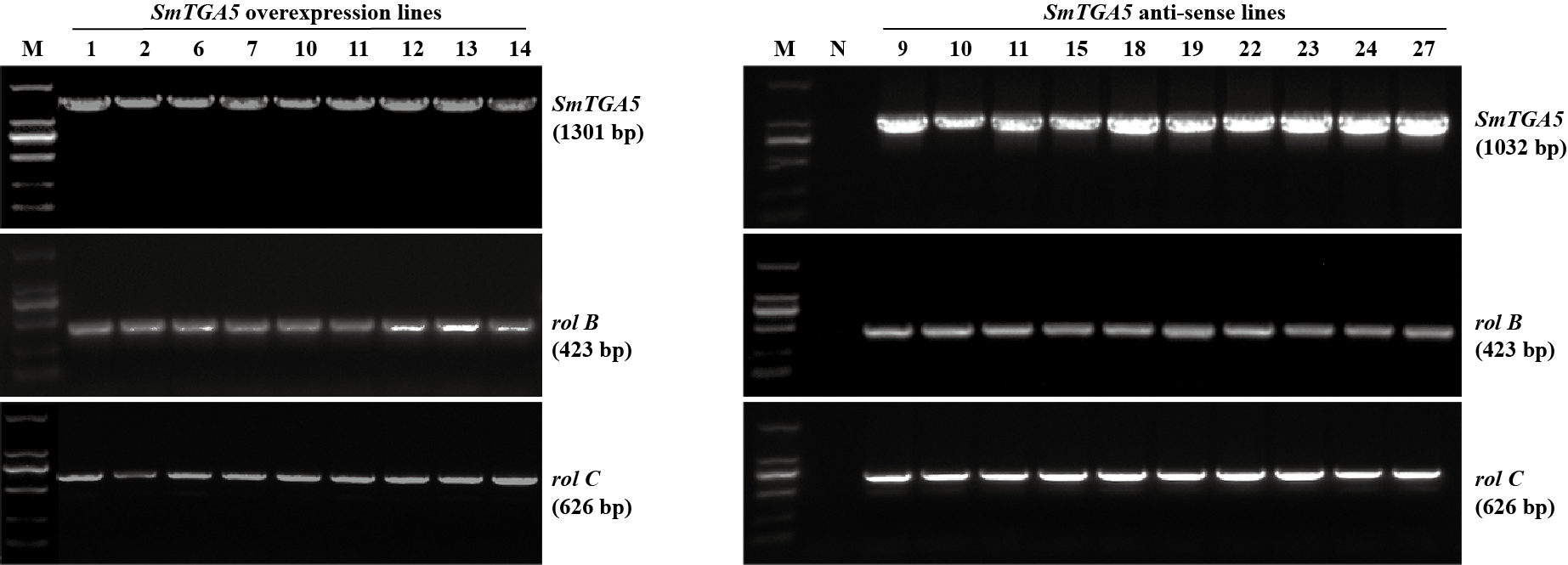

Supplement: Web_Material_uhad066 [file web_material_uhad066.zip › Fig. S7.tif]

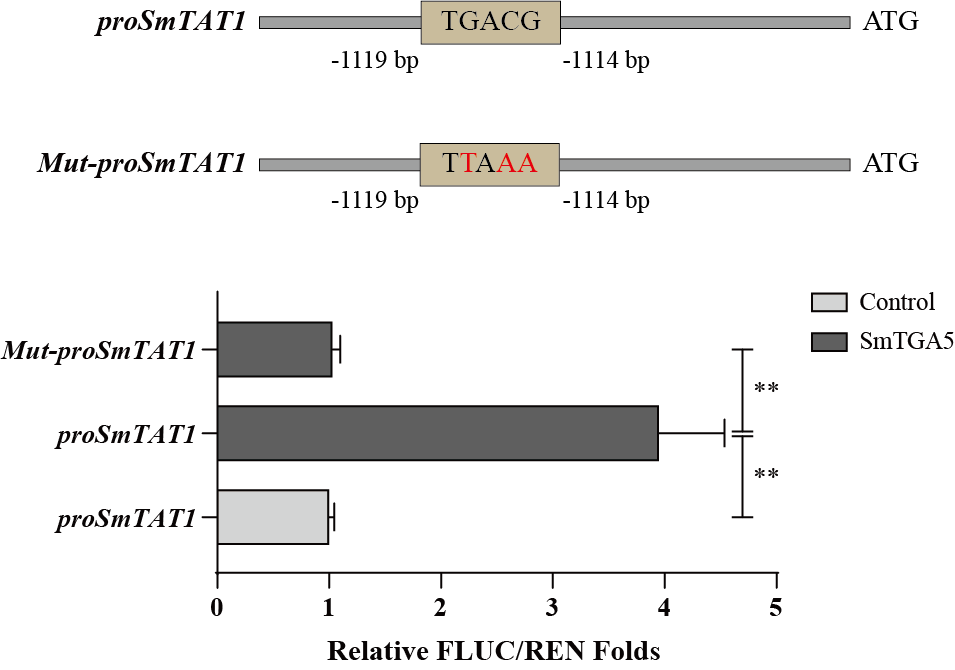

Supplement: Web_Material_uhad066 [file web_material_uhad066.zip › Fig. S8.tif]

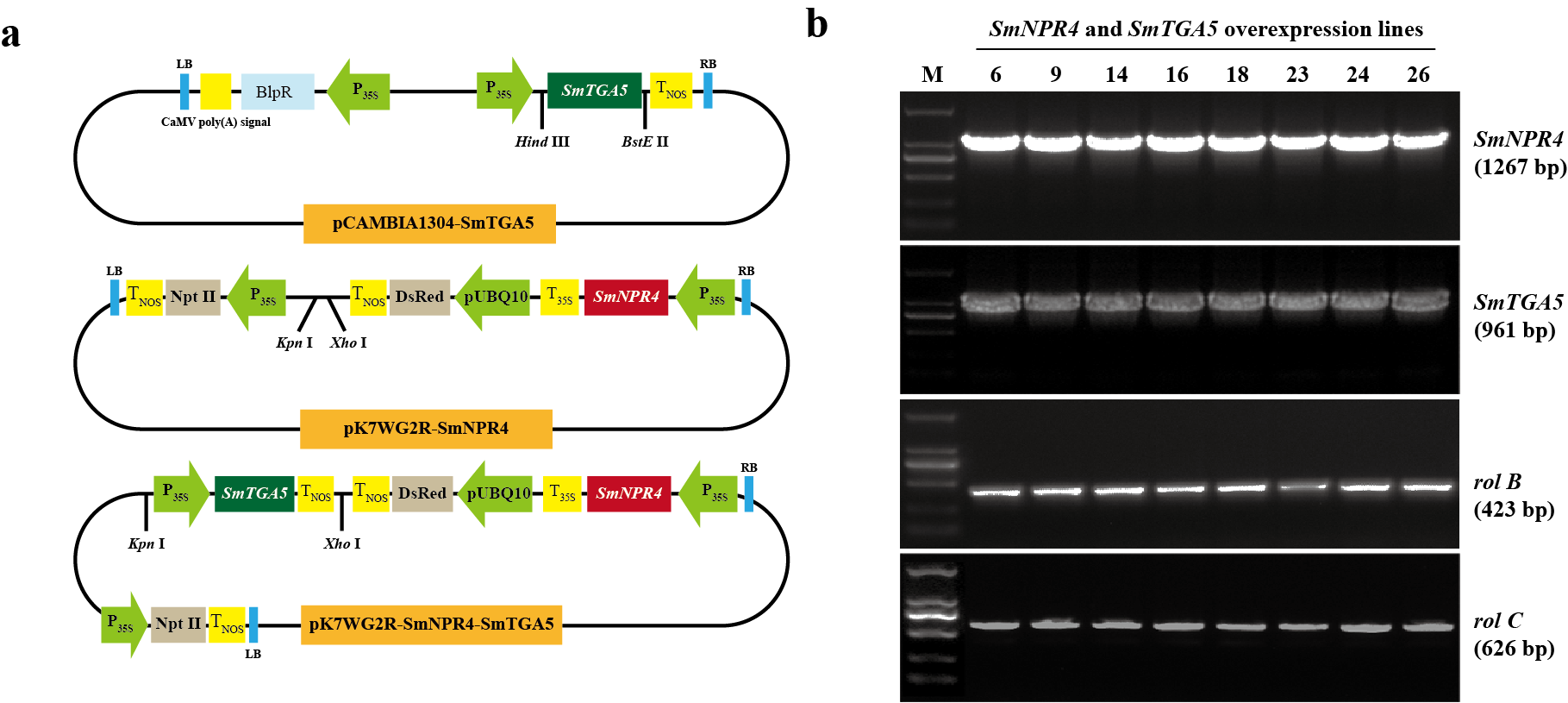

Supplement: Web_Material_uhad066 [file web_material_uhad066.zip › Fig. S9.tif]
